# Smart responsive Fe/Mn nanovaccine triggers liver cancer immunotherapy via pyroptosis and pyroptosis-boosted cGAS-STING activation

**DOI:** 10.1186/s12951-024-02354-2

**Published:** 2024-03-06

**Authors:** Qianying Du, Ying Luo, Lian Xu, Chier Du, Wenli Zhang, Jie Xu, Yun Liu, Bo Liu, Sijin Chen, Yi Wang, Zhigang Wang, Haitao Ran, Junrui Wang, Dajing Guo

**Affiliations:** 1 Department of Radiology, Second Affiliated Hospital of Chongqing Medical University, Chongqing, 400010 China; 2 Chongqing Key Laboratory of Ultrasound Molecular Imaging & Department of Ultrasound, Second Affiliated Hospital of Chongqing Medical University, Chongqing, 400010 China

**Keywords:** Bimetallic nanovaccine, Pyroptosis, cGAS-STING, Hepatocellular carcinoma, Magnetic resonance imaging

## Abstract

**Background:**

The prognosis for hepatocellular carcinoma (HCC) remains suboptimal, characterized by high recurrence and metastasis rates. Although metalloimmunotherapy has shown potential in combating tumor proliferation, recurrence and metastasis, current apoptosis-based metalloimmunotherapy fails to elicit sufficient immune response for HCC.

**Results:**

A smart responsive bimetallic nanovaccine was constructed to induce immunogenic cell death (ICD) through pyroptosis and enhance the efficacy of the cGAS-STING pathway. The nanovaccine was composed of manganese-doped mesoporous silica as a carrier, loaded with sorafenib (SOR) and modified with MIL-100 (Fe), where Fe^3+^, SOR, and Mn^2+^ were synchronized and released into the tumor with the help of the tumor microenvironment (TME). Afterward, Fe^3+^ worked synergistically with SOR-induced immunogenic pyroptosis (via both the classical and nonclassical signaling pathways), causing the outflow of abundant immunogenic factors, which contributes to dendritic cell (DC) maturation, and the exposure of double-stranded DNA (dsDNA). Subsequently, the exposed dsDNA and Mn^2+^ jointly activated the cGAS-STING pathway and induced the release of type I interferons, which further led to DC maturation. Moreover, Mn^2+^-related T1 magnetic resonance imaging (MRI) was used to visually evaluate the smart response functionality of the nanovaccine.

**Conclusion:**

The utilization of metallic nanovaccines to induce pyroptosis-mediated immune activation provides a promising paradigm for HCC treatment.

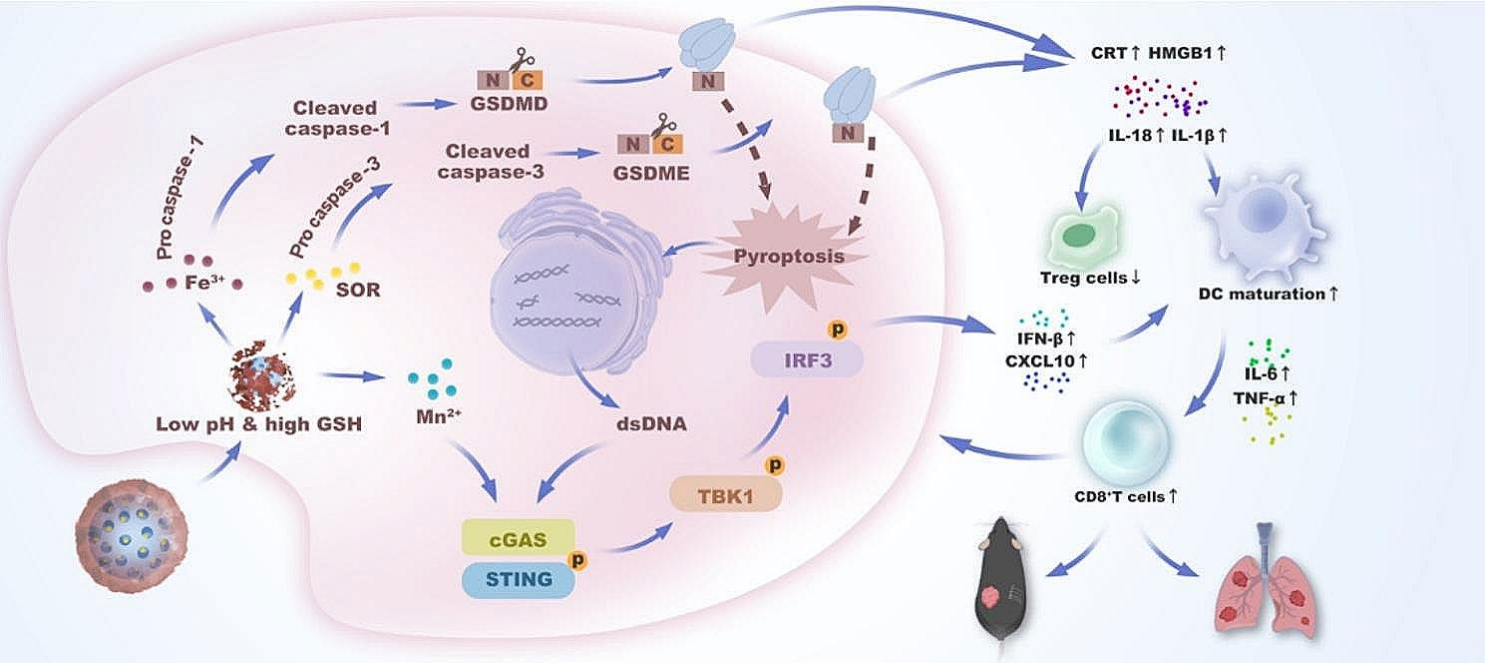

**Supplementary Information:**

The online version contains supplementary material available at 10.1186/s12951-024-02354-2.

## Introduction

Limited by current diagnostic techniques, hepatocellular carcinoma (HCC) is frequently detected at an advanced stage, thereby exacerbating the propensity for recurrence and metastasis [1]. Conventional treatment approaches such as surgery, radiotherapy and interventional therapy have been ineffective [2]. In recent years, immunotherapy has garnered considerable attention in combating tumor recurrence and metastasis. However, the limited immunogenicity of HCC restricts the responsiveness of patients to immunotherapy [3]. 

Fortunately, the potential of metal-based agents for tumor immunomodulation (metalloimmunotherapy) has been recognized, and these metal ions (Fe, Cu and Mn) have been shown to induce immunogenic cell death (ICD) in multiple tumor cells (including those of breast cancer, HCC, and colorectal cancer), [4] thereby leading to in situ vaccines. This intricate mechanism involves the release of damage-associated molecular patterns (DAMPs) and proinflammatory cytokines (IL-18, IL-1β, etc.), which endow immature DCs with the ability to capture tumor-associated antigens (TAAs), process antigens into peptides, and subsequently undergo maturation. Afterward, mature DCs efficiently present peptide-major histocompatibility complex (MHC) complexes to naive T cells, triggering the expansion and specialization of T cells [5]. Nonetheless, in conventional metal-mediated tumor apoptosis, it is difficult to achieve satisfactory antitumor immune activation due to the slow or even lack of release of DAMPs and proinflammatory cytokines [6]. Hence, developing metal-based high-immunogenicity antitumor strategies that can act as a substitute for apoptosis is urgently needed.

Pyroptosis, an inflammatory form of programmed cell death that is characterized by caspase activation, gasdermin (GSDM) cleavage, cell membrane perforation, and the rapid release of DAMPs and proinflammatory cytokines [7–9]. Ploetz et al. reported that Fe^3+^ can serve as a pyroptosis inducer, specifically through the caspase-1-mediated classical pyroptosis pathway [10–12]. Wang et al. further revealed that sorafenib (SOR), a conventional chemotherapeutic agent for HCC, activates the nonclassical pyroptosis pathway mediated by caspase-3 [13, 14]. Moreover, the abundant release of DAMPs and proinflammatory cytokines triggered by pyroptosis has been demonstrated to effectively stimulate the immune system [15]. These findings indicate that the combination of Fe^3+^ with SOR likely synergistically enhances both the classical and nonclassical pyroptosis pathways, thereby inducing a more thorough pyroptotic response and promoting enhanced immune activation. Interestingly, pyroptosis is often accompanied by DNA damage, [7, 9] including the production of double-stranded DNA (dsDNA). dsDNA can be detected by the cytoplasmic cGAS enzyme, leading to the conversion of adenylate triphosphate and guanosine triphosphate into 2’,3’-cyclic GMP-AMP and ultimately activating STING, resulting in the generation of type I interferons (especially IFN-β) and promoting the maturation of DCs [16–19]. Sun et al. confirmed that Mn^2+^ induces a hyperactivation of the cGAS-STING pathway, significantly enhancing the sensitivity of the cGAS enzyme to dsDNA [20]. To our knowledge, while previous studies have primarily relied on physical methods such as radiotherapy to induce dsDNA generation and activate the cGAS-STING pathway, the activation of this pathway through pyroptosis remains unexplored. Studying the inhibition of HCC recurrence and metastasis via pyroptosis-related immunomodulatory functions will be meaningful.

Despite the promising potential of these biological mechanisms, Fe^3+^, Mn^2+^, and SOR have distinctive pharmacokinetics in the body, which limits their accumulation at tumor sites in a uniform manner. Thankfully, with the rapid advancement of nanomedicine, nanoparticles (NPs) have emerged as a promising strategy for achieving the synchronous infiltration of metal ions and drugs into tumor tissues through the enhanced permeability and retention (EPR) effect [21, 22]. Another aspect to consider is that proteins in the gasdermin family are ubiquitously expressed in normal tissues, and nonspecific activation of the pyroptosis pathway in these tissues can initiate deleterious inflammatory responses, causing irreversible tissue damage [23]. To ensure biosecurity, these NPs must also possess advanced functionalities such as smart responsive release and imaging guidance within the unique tumor microenvironment (TME).

**Scheme 1:**
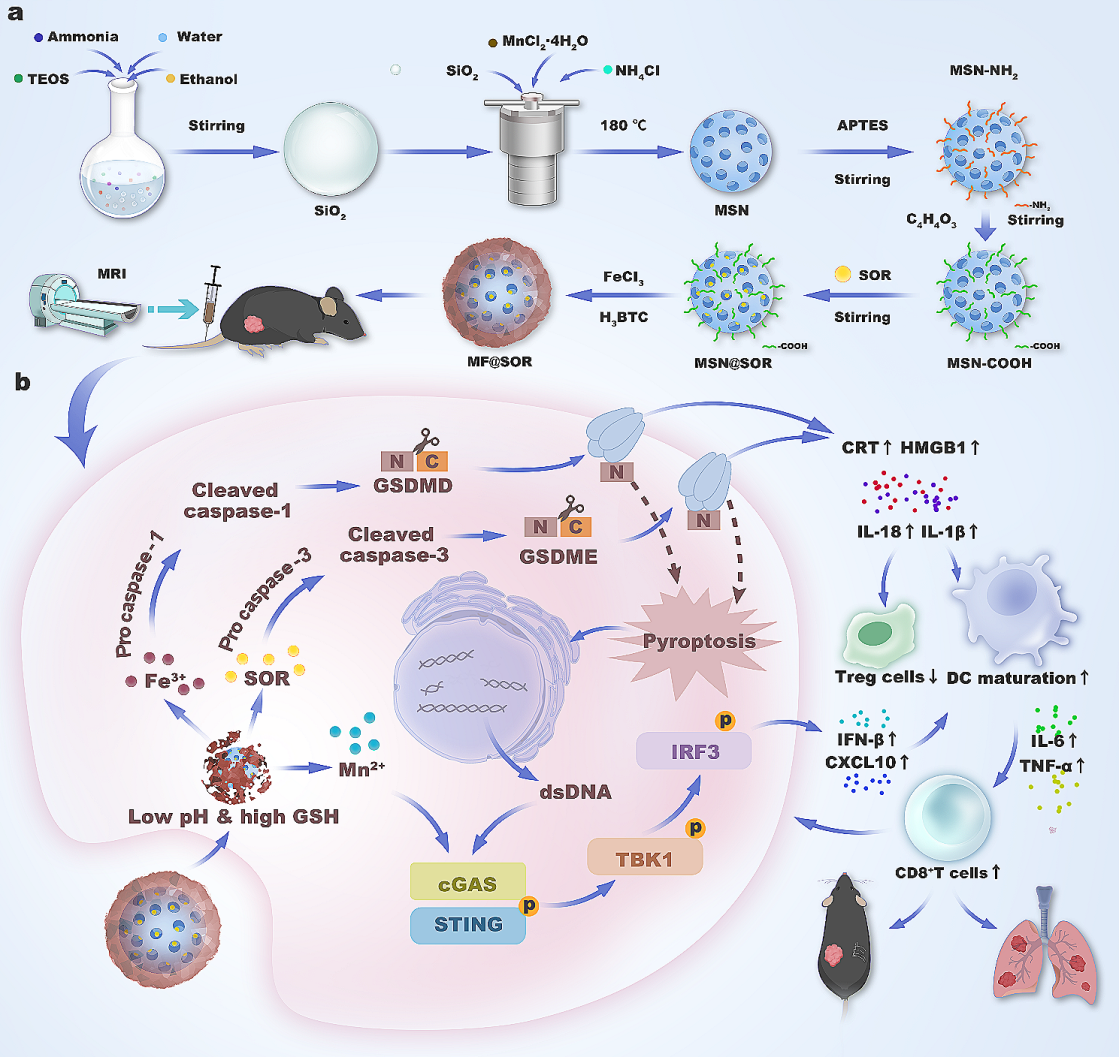
Schematic illustration of the synthesis **(a)** and mechanism **(b)** of the nanovaccine in liver cancer immunotherapy

Therefore, by employing a hydrothermal method to perforate solid silicon dioxide (SiO_2_) and dope it with manganese, we constructed Mn-doped mesoporous silica nanoparticles (MSN). Due to the formation of abundant -Mn-O- bonds in the aforementioned process, these MSN emerged as smart responsive candidates for manganese delivery and T1 magnetic resonance imaging (MRI), selectively releasing Mn^2+^ through the cleavage of -Mn-O- bonds within the TME with a low pH and high glutathione (GSH) concentration [24–28]. In this research, to endow the NPs with superior immune activation functionality, we reconstructed the NPs by altering the proportions of the ingredients, followed by loading SOR and coating them with MIL-100 (Fe) to generate a metallic nanovaccine (MF@SOR, Scheme 1a). First, due to its nanoscale size and strong EPR effects, [22] MF@SOR passively targeted and aggregated in the tumor region through incomplete tumor vessel walls. Upon engulfment by tumor cells, MF@SOR responded to the intratumoral environment at a low pH and a high GSH level, after which the Fe^3+^ detached from the MIL-100 (Fe) coating and, in synergy with SOR, induced powerful pyroptosis. This resulted in the release of an abundance of immunogenic factors, promoting DC maturation and the exposure of dsDNA. Sequentially, leveraging the exposed dsDNA, the typical antitumor immunostimulatory signaling pathway cGAS-STING was activated from the start and further amplified by the Mn^2+^ that dissociated from the nanovaccine, leading to the generation of type I IFN and subsequent DC maturation. Under the influence of inflammatory factors and the synergistic effect of mature DCs, there was a notable increase in the population of cytotoxic T lymphocytes (CTLs) concomitant with a reduction in the abundance of regulatory T cells (Tregs), resulting in remodeling of the immunosuppressive microenvironment within the tumor milieu. Furthermore, some memory immune cells were activated, and these immunomodulatory functions collectively suppressed tumor recurrence and metastasis (**Scheme 1b**). In brief, MF@SOR accomplished the synchronized delivery of multiple components (Fe, Mn, and SOR) and imaging monitoring through smart response to the conditions of the TME. Additionally, MF@SOR effectively promoted immune therapy for HCC through synergistic activation of the pyroptosis and pyroptosis-boosted cGAS-STING pathway.

## Methods

### Materials

Tetraethyl orthosilicate (TEOS) (T819505), trimesic acid (H_3_BTC) (T819407), iron(III) chloride (FeCl_3_) (I811935), succinic anhydride (S817605), (3-aminopropyl)triethoxysilane (APTES) (A800523), NH_4_Cl (A801305), N,N-dimethylformamide (DMF) (N807505) and ammonium hydroxide solution (NH_3_·H_2_O, 28%) were purchased from Macklin (Shanghai, China). Manganese chloride (MnCl_2_·4H_2_O) (R000534) was obtained from Rhawn (Shanghai, China). Fetal bovine serum (FBS) was obtained from Cell-Box (HK) Biological Products Trading Co., Ltd. Dulbecco’s modified Eagle’s medium (DMEM) (PYG0074), 5% BSA confining liquid (BSA) (AR0004), and phosphate-buffered saline (PBS) (PYG0021) were purchased from Boster (Wuhan, China). Penicillin–streptomycin (C0222), trypsin-EDTA solution (C0201), the DNA Damage Assay Kit by phosphorylated H2AX histone (γ-H2AX) immunofluorescence (IF), and DiI (C1036) were purchased from Beyotime Biotechnology (Jiangsu, China). Enzyme-linked immunosorbent assay (ELISA) kits, including those for IL-6 (MM-0163M1), IFN-β (MM-0124M1), TNF-α (MM-0132M2), IL-18 (MM-0169M1), and IL-1β (MM-0040M1), were purchased from MEIMIAN (Jiangsu, China). Calcein-AM/PI Double Staing Kit (C542) was obtained from Dojindo Laboratories (Kumamoto, Japan). SOR (GC17369) and Cell Counting Kit-8 (CCK-8) (GK10001) were purchased from GLPBIO (Montclair, CA, USA).

### Synthesis of various NPs

#### Synthesis of SiO_2_ NPs

Silicon dioxide NPs were prepared employing the approach outlined in a prior investigation [24]. Briefly, a solution was prepared by combining 98 mL of ethanol with 10 mL of water. To this system, 1.25 mL of NH_3_·H_2_O (28%) and 5 mL of TEOS were added. The blend was agitated at a speed of 300 rpm for a duration of 20 h at ambient temperature. Subsequently, the SiO_2_ NPs were collected by centrifugation, followed by three washes with ethanol and water.

#### Synthesis of MSN

First, MnCl_2_·4H_2_O (80 mg) and NH_4_Cl (400 mg) were dissolved in 77.4 mL of deionized water. Next, the above SiO_2_ NP aqueous solution (1 mL, 80 mg mL ^− 1^) and NH_3_·H_2_O (1.6 mL, 28%) were added into the system. Following that, the mixture was heated at 180 °C for 10 h. Then, the MSN were separated through centrifugation.

#### Synthesis of MSN@SOR

First, the carboxyl radical was modified on the MSN surface by the following method. Forty milligrams of MSN were dispersed in an edmixture solution consisting of 20 mL of ethanol and 400 µL of APTES. Then, the mixture was agitated, and MSN-NH_2_ was obtained after washing and collection by centrifugation. The next step was to carboxylate the amino radical, and to do this, 40 mg of MSN-NH_2_ was first decentralized in 15 mL of ethanol. Then, 140 mg of succinic anhydride (C_4_H_4_O_3_) was fully dissolved in DMF and added into the aforementioned ethanol solution. After being stirred for 12 h at ambient temperature, the MSN-COOH were gathered through centrifugation, and subsequently rinsed multiple times with ethanol. In order to obtain MSN@SOR, a dispersion of MSN-COOH (8 mg) in 5 mL of ethanol containing SOR (3.5 mg) was prepared. The mixture was then gently stirred for a duration of 12 h. Afterward, the mixture was subjected to centrifugation, and the resulting precipitate was collected and washed three times to eliminate unloaded SOR.

#### Synthesis of MF@SOR

To apply a layer of MIL-100 (Fe) onto the exterior of the MSN@SOR, the as-prepared MSN@SOR aqueous solution (20 mL, 1 mg mL^− 1^) was mixed with an ethanol solution of FeCl_3_ (10 mL, 8.1 mg mL^− 1^) with vigorous stirring for 15 min. Then, an ethanol solution of H_3_BTC (10 mL, 10.5 mg mL^− 1^) was immersed into the precursor solution for 2 h of reaction at 40 °C. Subsequently, the resulting product was isolated through centrifugation and subjected to multiple washes using ethanol and water. Finally, the product was preserved at a temperature of 4 °C until it was ready for subsequent utilization. MF was synthesized according to the above method without the addition of SOR. DiI- and DiR-labeled MF were prepared by introducing DiI or DiR into the aqueous solution of MF under continuous stirring. (250 rpm, 12 h).

### Characterization of different NPs

Transmission electron microscopy (TEM) (Talos F200X, USA) and scanning electron microscopy (SEM) (HITACHI S4800, Japan) were utilized to observe the morphological characteristics of the synthesized NPs. Following TEM analysis, the elemental maps of Mn, Si, O, and Fe were also investigated. Zetasizer Nano ZS90 was used to analyze diameters and zeta potentials of various NPs. To examine the stability of the NPs, MF@SOR were dispersed in deionized water, saline, FBS, and DMEM, and the particle size was analyzed at various time intervals (1, 3, 5, 7, 9, 11, and 13 days). The X-ray diffraction (XRD) pattern was obtained with an X-ray diffractometer (BRUKER D8 ADVANCE, Germany). Brunauer–Emmett–Teller (BET) analysis was conducted with a Quantachrome Autosorb IQ3 apparatus (USA). The compositions of the MF@SOR were verified by Fourier transform infrared (FTIR) spectroscopy (iS10 FT-IR spectrometer, USA). To assess the encapsulation efficiency (EE) and loading capacity (LC) of SOR in MF@SOR, a Shimadzu LC-2010AHt instrument from Japan was utilized for high-performance liquid chromatography (HPLC) analysis. X-ray photoelectron spectroscopy (XPS) of the MF@SOR NPs was also performed (Thermo Scientific K-Alpha, USA). Inductively coupled plasma‒optical emission spectrometry (ICP‒OES) (Thermo ICP‒OES 7200; USA) was used to evaluate the EE values of Fe and Mn in the MF@SOR.

### In vitro drug release kinetics

To verify the pH- and GSH-responsive characteristics of MF@SOR, MF@SOR (4 mg mL^− 1^) was suspended in four different PBS-containing media with the following conditions: (1) pH 7.4; (2) pH 7.4, 10 mM GSH; (3) pH 6.5, 10 mM GSH; and (4) pH 5.0, 10 mM GSH, followed by mild stirring for 48 h (220 rpm, 37 °C). At predetermined time points (10 and 30 min, and 1, 2, 4, 6, 8, 12, 24, and 48 h), the concentration of SOR in different media was detected using HPLC.

### In vitro MRI

To investigate the in vitro MRI characteristics of MF@SOR under diverse conditions ((1) pH 7.4; (2) pH 6.5; (3) pH 5.0; (4) pH 7.4, 10 mM GSH; (5) pH 6.5, 10 mM GSH; and (6) pH 5.0, 10 mM GSH), a 3.0 T MRI scanner (MAGNETOM Prisma, Siemens, Germany) was used to scan NPs at various concentrations (Mn: 0.08, 0.016, 0.033, 0.066, and 0.132 mM). The transverse relaxivity (r1) value was determined by performing a linear regression analysis of the inverse T1 relaxation time against the Mn concentration.

### Cell experiments

#### Cell Culture

Human umbilical vascular endothelial cells (HUVECs) and Hepa1-6 cells were obtained from Boster (Wuhan, China), while JAWS II cells were obtained from Meisen (Zhejiang, China). HUVECs and Hepa1-6 cells were grown in DMEM containing 10% FBS and 1% penicillin‒streptomycin solution. JAWS II cells were grown in RPMI-1640 medium supplemented with 20% FBS, 5 ng mL^− 1^ granulocyte-macrophage colony stimulating factor (GM-CSF), and 1% penicillin‒streptomycin solution. To maintain cell viability, the cells were cultured in a CO_2_ incubator at 37 °C with a 5% CO_2_ atmosphere.

#### Intracellular endocytosis of MF@DiI

First, Hepa1-6 cells (1 × 10^5^ cells per dish) were individually seeded in confocal dishes and incubated overnight. Subsequently, the culture medium was substituted with DMEM supplemented with MF@DiI (200 µg mL^− 1^) for 30 min or 1, 2, 3, or 4 h of coincubation. Afterward, the cells were fixed with 4% paraformaldehyde (1.5 mL) for 15 min. Next, 100 µL of DAPI was used to counterstain the nuclei for 15 min. Finally, the uptake of MF@DiI was visualized using confocal laser scanning microscopy (CLSM) (Nikon, Tokyo, Japan).

Flow cytometry (FCM) (FACS Vantage SE, Becton Dickinson, San Jose, CA, USA) was applied to quantitative analyze the intracellular uptake of NPs. Hepa1-6 cells were planted into 6-well plates and incubated overnight. Subsequently, FCM was used to analyze cells which were incubated with MF@DiI for various durations.

#### In vitro cytotoxicity evaluation

Hepa1-6 cells and HUVECs were seeded in 96-well plates overnight and then incubated for 12 h with various concentrations of MSN, MSN@SOR, MF or MF@SOR (20, 40, 80, 120, 160 and 200 µg mL^− 1^). Additionally, the toxicity of pure SOR to Hepa1-6 cells was assessed by treating the cells with a gradient of concentrations of SOR (10, 20, 30, 40, 50, and 60 µg mL^− 1^). Finally, cell viability was assessed using the CCK-8 assay.

#### In vitro antitumor effect

Hepa1-6 cells were plated in 6-well plates and incubated overnight and subjected to treatment of DMEM, SOR, MSN, MSN@SOR, MF, or MF@SOR (SOR at 30 µg mL^− 1^, MSN, MSN@SOR, MF, and MF@SOR at 200 µg mL^− 1^) for 12 h, and the live and dead cells were stained with Calcein-AM and PI respectively. The antitumor effects after different treatments were qualitatively analyzed with an inverted fluorescence microscope.

#### Evaluation of pyroptosis and DC maturation in vitro

To study the release of calreticulin (CRT) and high mobility group protein B1 (HMGB1) and DNA damage induced by the NPs, first, hepa1-6 cells were cultured in separate confocal dishes and incubated for 12 h. Afterward, the cells were treated with DMEM, SOR, MSN, MSN@SOR, MF, MF@SOR or MF + SOR (SOR at 30 µg mL^− 1^, MSN, MSN@SOR, MF, and MF@SOR at 200 µg mL^− 1^) for 12 h, washed with PBS three times, and fixed in 4% paraformaldehyde solution (1.5 mL) for a quarter of an hour. Next, cells were blocked with 5% BSA for 45 min, and incubated with CRT, HMGB1 or γ-H2AX polyclonal antibodies at 4 °C overnight. After washing with PBS 3 times, the cells were incubated with corresponding fluorescent antibodies for 1 h. Finally, the nuclei were counterstained with DAPI, and then the stained dishes were subsequently imaged via CLSM.

To examine the morphology of hepa1-6 cells, the cells were seeded into 6-well plates. Next, the cells were incubated with DMEM, SOR, MSN, MSN@SOR, MF, or MF@SOR (SOR at 30 µg mL^− 1^, MSN, MSN@SOR, MF, and MF@SOR at 200 µg mL^− 1^) for 12 h. The cell morphology was observed using an inverted fluorescence microscope.

For the ELISAs, the cells were treated as described for morphological observation; afterward, the culture supernatant was collected and then analyzed with reagents of the ELISA kits for IL-18 and IL-1β according to the manufacturer’s instructions.

For in vitro DC maturation experiments, Transwell plates (LABSELECT 14,111, China) were used to coincubate JAWS II cells and drug-retreated hepa1-6 cells. JAWS II cells were cultured in the lower compartment of the Transwell system overnight at 37 °C, and hepa1-6 cells were seeded into the upper compartment and treated as mentioned above with DMEM, SOR, MSN, MSN@SOR, MF, or MF@SOR (SOR at 30 µg mL^− 1^, MSN, MSN@SOR, MF, and MF@SOR at 200 µg mL^− 1^) for 4 h. Following a 24-hour period of coincubation, the JAWS II cells were collected and stained with antibodies. FCM was employed to assess the percentage of mature DCs. The proinflammatory cytokines (IFN-β, CXCL10, IL-6 and TNF-α) in the supernatant were detected by using ELISA kits following a standard protocol. Moreover, immature and mature DCs were photographed with an inverted fluorescence microscope.

### Animal experiment

#### Establishment of Syngeneic HCC Model mice

All animal experiments were conducted in compliance with the regulations set by the Animal Ethics Committee of Chongqing Medical University and Institutional Animal Care guidelines (No. (2022) 224). Male C57BL/6J mice were sourced from the Experimental Animal Center of Chongqing Medical University. To establish subcutaneous syngeneic C57BL/6J HCC mouse model, 5 × 10^6^ Hepa1-6 cells in 75 µL of PBS were administered via subcutaneous injection into the right flank of the mice (5 mice were in each group).

#### In vivo antitumor mechanism

A subcutaneous hepa1-6 tumor model was established 7 days after tumor cell injection, and these mice were then randomly split into 5 groups to receive saline, MSN (45 mg kg^− 1^), SOR (30 mg kg^− 1^), MSN@SOR (45 mg kg^− 1^) or MF@SOR (45 mg kg^− 1^) on the 1st, 4th, and 8th days. On day 9, the tumor tissue was amputated and fixed in 4% polyformaldehyde. Subsequently, immunofluorescence (IF) staining for CRT, HMGB1, p-STING, p-TBK1 and p-IRF3 was performed, and cleaved caspase-1 and cleaved caspase-3 were stained via immunohistochemistry (IHC). All images were analyzed using ImageJ software. Moreover, mouse serum was collected for ELISAs to detect IL-18, IL-1β, CXCL10, TNF-α, IFN-β and IL-6 levels.

#### In vivo immunomodulation

For immune cell analysis, tumors, tumor-draining lymph nodes (TDLNs), and spleens were collected from C57BL/6J mice in different groups 8 days after the initial treatment. The tumors and TDLN tissues were subjected to digestion for 40 min at 37 °C using 1 mL of digestion solution. Subsequently, red blood cell lysis buffer was employed to remove red blood cells, after which the tissues were filtered through 40 μm cell strainers to obtain single cells. The spleens were also ground and filtered using 70 μm cell strainers. Finally, all samples were stained with corresponding antibodies and analyzed by FCM. Furthermore, tumors from each group were excised, fixed and sectioned to analyze the expression of Foxp3^+^ using IHC, and the infiltration of CD8^+^ T cells was assessed through IF staining. Quantification of Foxp3^+^ expression and the CD8^+^ T-cell signals was performed using ImageJ software.

#### In vivo antitumor effects

Considering the excellent antitumor efficacy of MF@SOR in vivo, the therapeutic effects of these NPs were further evaluated. A subcutaneous tumor model was established in C57BL/6J mice and the same treatments were administered using the aforementioned methods. The tumor volume was calculated using the following formula: volume = width ^2^ × length/2. The treatments were administered for 14 days. Subsequently, the tumor tissues were fixed and hematoxylin and eosin (H&E) staining was performed, along with IF staining for terminal deoxynucleotidyl transferase dUTP nick end labeling (TUNEL) and proliferating cell nuclear antigen (PCNA). The major organs were carefully removed and fixed for subsequent H&E staining. All the images were analyzed using ImageJ software. Additionally, survival analysis was conducted, and the mice were observed for a period of 60 days. The mice were humanely euthanized once their tumor volume reached 1500 mm^3^.

#### In vivo MRI

For the in vivo MRI experiment, the MRI performance of the MF@SOR was investigated in the subcutaneous HCC model. After intravenous injection of MF@SOR (45 mg kg^− 1^), T1 MRI of the tumor area was performed at various time intervals (0, 2, 4, 8, 12 and 20 h).

#### Long-term immune effects in the recurrent and metastasis models

To construct the recurrent tumor model, a primary subcutaneous model was established using the aforementioned methods and the same treatments were administered using the aforementioned methods. On day 15, the primary tumor located in the right flank of each mouse was surgically excised. Subsequently, all the mice were allowed to recover for 5 days. On day 20, rechallenge was conducted by injecting 5 × 10^6^ Hepa1-6 cells into the opposite flanks of the mice. The growth of the rechallenged tumors and changes in body weight were monitored on alternate days until day 40. The tumor volume was calculated using the aforementioned formula. On the 40th day, the mice were euthanized, tumors and spleens were prepared as single-cell suspensions. The cells were then stained with corresponding antibodies. Finally, the proportions of various cells were analyzed via FCM.

To create the distant metastasis tumor model, primary subcutaneous models were established using previously mentioned procedure. Seven days after tumor cell injection, the mice were categorized into five groups for treatment as described above. Then, on day 10, the mice in each group were intravenously reinjected with hepa1-6 cells (1 × 10^6^). Furthermore, on day 20, the mice in each group were euthanized, and their lung tissue was extracted, fixed, and stained with Bouin’s solution to observe lung metastases in mice from different groups.

#### Tumor accumulation, biodistribution, and clearance rates of MF@SOR

ICP-MS and in vivo fluorescence imaging (FLI) were used to analysis the biodistribution and tumor accumulation of MF@SOR. MF@DiR were intravenously injected into tumor-bearing mice. Then, FLI and ICP-MS were performed to observe tumor accumulation at certain time intervals (0, 2, 4, 8, 24, 48 and 72 h) postinjection, after which the mice were sacrificed, and the tumors and major organs were extracted for biological distribution analyses. Clearance rates of MF@SOR were validated by measuring the manganese ion concentrations in the blood at different time points after MF@SOR injection through ICP-MS.

#### Biosafety of the MF@SOR

The in vivo biosafety of the MF@SOR was assessed in male Kunming mice aged 6–8 weeks. The mice were allocated into six groups using a randomization process, including a control group and groups that were euthanized at 1, 3, 7, 14, and 21 d after NPs injection. Each mouse received a dose of 200 µL of MF@SOR at a concentration of 4.5 mg mL^− 1^. At the predetermined time points, the mice were euthanized. Routine blood and biochemical examinations were conducted using blood samples collected, while major organs were excised for histological analysis using H&E staining.

### Statistical analysis

GraphPad 9.5.1 (La Jolla, CA, USA) was utilized for statistical analysis. All the quantitative data are presented as the mean ± standard deviation (SD). Unpaired multiple t tests and analysis of variance (ANOVA) were used to examine differences among multiple groups. The statistical tests were two-sided, and values with *P* < 0.05 were considered to indicate statistical significance.

## Results and discussion

### Characterization of different NPs

**Fig. 1:**
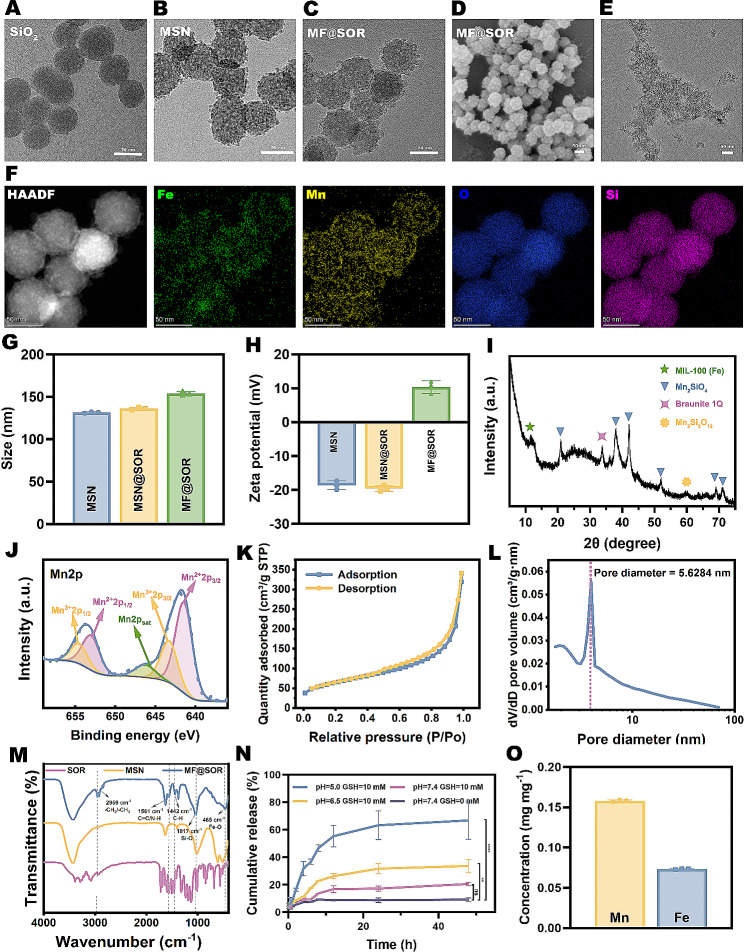
Characterization of the different NPs. TEM images of the different NPs: **(A)** SiO_2_, **(B)** MSN, and **(C)** MF@SOR (scale bar: 50 nm). **(D)** SEM image of the MF@SOR (scale bar: 50 nm). **(E)** TEM image of MF@SOR in response to an environment at pH = 5 with GSH (scale bar: 50 nm). **(F)** HAADF and elemental mapping of MF@SOR (scale bar: 50 nm). **(G)** Diameters and **(H)** zeta potentials of the different NPs (*n* = 3). **(I)** XRD patterns of the MSN. **(J)** XPS spectrum of Mn in the MF@SOR. **(K)** N_2_ adsorption-desorption isotherm and **(L)** pore size distribution of the MSN. **(M)** FTIR spectra of SOR, MSN and MF@SOR. **(N)** The release of SOR from MF@SOR in PBS at pH 5.0 with GSH, pH 6.5 with GSH, pH 7.4 with GSH and pH 7.4 (*n* = 3). **(O)** The contents of Fe and Mn in the MF@SOR measured by ICP‒OES. In panel **(N)**, the data are presented as the mean ± SD, and the *p* values were calculated by ANOVA. ∗ *p* < 0.05, ∗∗ *p* < 0.01, ∗∗∗ *p* < 0.001, ∗∗∗∗ *p* < 0.0001, ns, not significant

MF@SOR was prepared according to the steps shown in Scheme 1a. Representative photographs of the different NPs are shown in Figure S1. SiO_2_ NPs were prepared as precursors, and the TEM images revealed that these NPs were spherical and dispersed uniformly (Fig. 1A). The mesoporous structure of the MSN was clearly observed by TEM (Fig. 1B). The hydrodynamic size distribution of the obtained MSN was approximately 131.37 ± 0.58 nm (Fig. 1G), and their zeta potential was approximately − 18.63 ± 1.06 mV (Fig. 1H). After SOR was loaded, the overall size of the MSN@SOR increased to approximately 135.93 ± 1.35 nm (Fig. 1G), and its zeta potential changed to -19.57 ± 0.71 mV (Fig. 1H). Thus, these findings demonstrated that the loading of SOR caused minimal alterations in the diameter and zeta potential of the MSN. Finally, MSN@SOR was wrapped with MIL-100 (Fe) by physical stirring to obtain MF@SOR. TEM and SEM images of MF@SOR are shown in Fig. 1C & D. After coating with MIL-100 (Fe), the pores of the MSN were covered, the size of the NPs notably increased to approximately 153.87 ± 1.8 nm (Fig. 1G), and the zeta potential was reversed, changing to 10.31 ± 1.53 mV (Fig. 1H), indicating the successful incorporation of MIL-100 (Fe).

High-angle annular dark field (HAADF) analysis and elemental maps of Fe, Mn, Si and O verified the elemental composition of MF@SOR (Fig. 1F). Afterward, the MF@SOR were exposed to a low pH environment containing GSH, and the TEM image (Fig. 1E) revealed that the MF@SOR underwent a morphological change, losing their original shape and becoming fragmented. In addition, after being suspended in different solutions for different durations, the average hydrodynamic diameter of the prepared MF@SOR ranged from 150 to 170 nm, suggesting that the NPs exhibited favorable colloidal stability (Figure S2). The XRD pattern (Fig. 1I) of the as-prepared MF@SOR revealed the presence of braunite-1Q (Mn^2+^Mn_6_^3+^SiO_12_, JCPDS no. 89-5661) and other manganese silicate phases (Mn_2_SiO_4_, JCPDS no. 02-1327 and Mn_5_Si_3_O_12_, JCPDS no. 37–0221), confirming the covalent bonding of manganese species within the silica framework. The characteristic peak near 2θ = 11° was consistent with the standard pattern of MIL-100 (Fe), [29, 30] indicating successful MIL-100 (Fe) shell coating. The N_2_ adsorption-desorption curve further indicated that the as-synthesized MSN exhibited a type IV isotherm with a type H3 hysteresis loop at P/P 0 = 0.4-1.0, demonstrating that the MSN had a porous mesoporous structure (Fig. 1K). In addition, the average pore size of the MSN was 5.6284 nm (Fig. 1L), and the pore volume was 0.3213 cm³ g^− 1^, indicating that the MSN had the potential to carry SOR. FTIR spectroscopy (Fig. 1M) was applied to validate the successful formation of the MF@SOR. Overall, the infrared spectrum of MF@SOR retained the typical characteristic peaks of MSN, and the absorption peak at 1017 cm^− 1^ was a typical mesoporous silica Si-O asymmetric stretching vibration mode, revealing that the major structure of the synthesized material was MSN. Notably, multiple absorption peaks appeared near 2959 cm^− 1^ and were attributed to the C-H bonds in SOR. The absorption peaks near 1561 cm^− 1^ were associated with the N-H and C = C structures in the SOR structure, while the absorption peak near 1442 cm^− 1^ was correlated with the methyl stretching vibrations. The presence of these characteristic peaks confirmed the successful loading of SOR into the MSN. In addition, a sharp absorption peak was observed in the sample near 465 cm^− 1^, mainly due to the Fe-O bending vibration mode, verifying successful modification with MIL-100 (Fe). XPS was used to analyze the valence states of manganese (Fig. 1J) and iron (Figure S3). The manganese 2p3/2 spectrum was deconvoluted into two characteristic bands at 641.43 and 643.29 eV, which were indexed to Mn^2+^ and Mn^3+^, respectively; similarly, the iron 2p3/2 spectrum was deconvoluted into two characteristic bands at 710.19 and 711.84 eV, which were indexed to Fe^2+^ and Fe^3+^, respectively. ICP‒OES was used to evaluate the manganese and iron contents in MF@SOR, yielding values of 0.1578 ± 0.0009 mg mg^− 1^ and 0.073 ± 0.0004 mg mg^− 1^, respectively (Fig. 1O). Based on the HPLC analysis, the EE and LC of SOR were 35% and 10.7%, respectively. These characterizations confirmed the successful synthesis of the MF@SOR nanovaccine.

### In vitro drug release kinetics

As shown in Fig. 1N, approximately 65% of the total SOR was released from the MF@SOR after 48 h of incubation in PBS at pH 5.0 with 10 mM GSH. In PBS at pH 6.5 with 10 mM GSH, approximately 40% of the overall SOR was liberated. At pH 7.4 with 10 mM GSH, approximately 20% of the total SOR was released, while less than 10% of the total SOR was released at pH 7.4 after 48 h. Clearly, the release of SOR from MF@SOR increased gradually as the pH of the environment decreased with the help of GSH. These results indicate that the liberation of SOR was greater in acidic and reducing environments than in neutral conditions.

### In vitro MRI

Owing to its excellent spatial resolution and ability to penetrate deep tissues, MRI has emerged as a highly popular imaging technique for clinical diagnosis. In an environment with a low pH or high GSH content, the T1 MRI contrast signal increased with increasing NP concentration (Figure S4). However, the observed increase in the T1 MRI contrast signal was less pronounced in the physiological environment (pH 7.4). Additionally, a clear linear relationship was observed between the 1/T1 values and the concentration of Mn (Figure S5). In comparison to those in the pH 7.4 group, the r_1_ values in the other groups increased remarkably, and the r_1_ value in the pH 5.0 + 10 mM GSH group reached 54.677 mM^− 1^S^− 1^, which was probably caused by the degradation of MIL-100 (Fe) under acidic conditions and the inner Mn^2+^ was dissociated from the MSN complex in the reducing environment induced by GSH, demonstrating that MF@SOR can be used as TME stimuli-responsive T_1_ MRI contrast agent.

### Cell experiments

#### Intracellular endocytosis

**Fig. 2:**
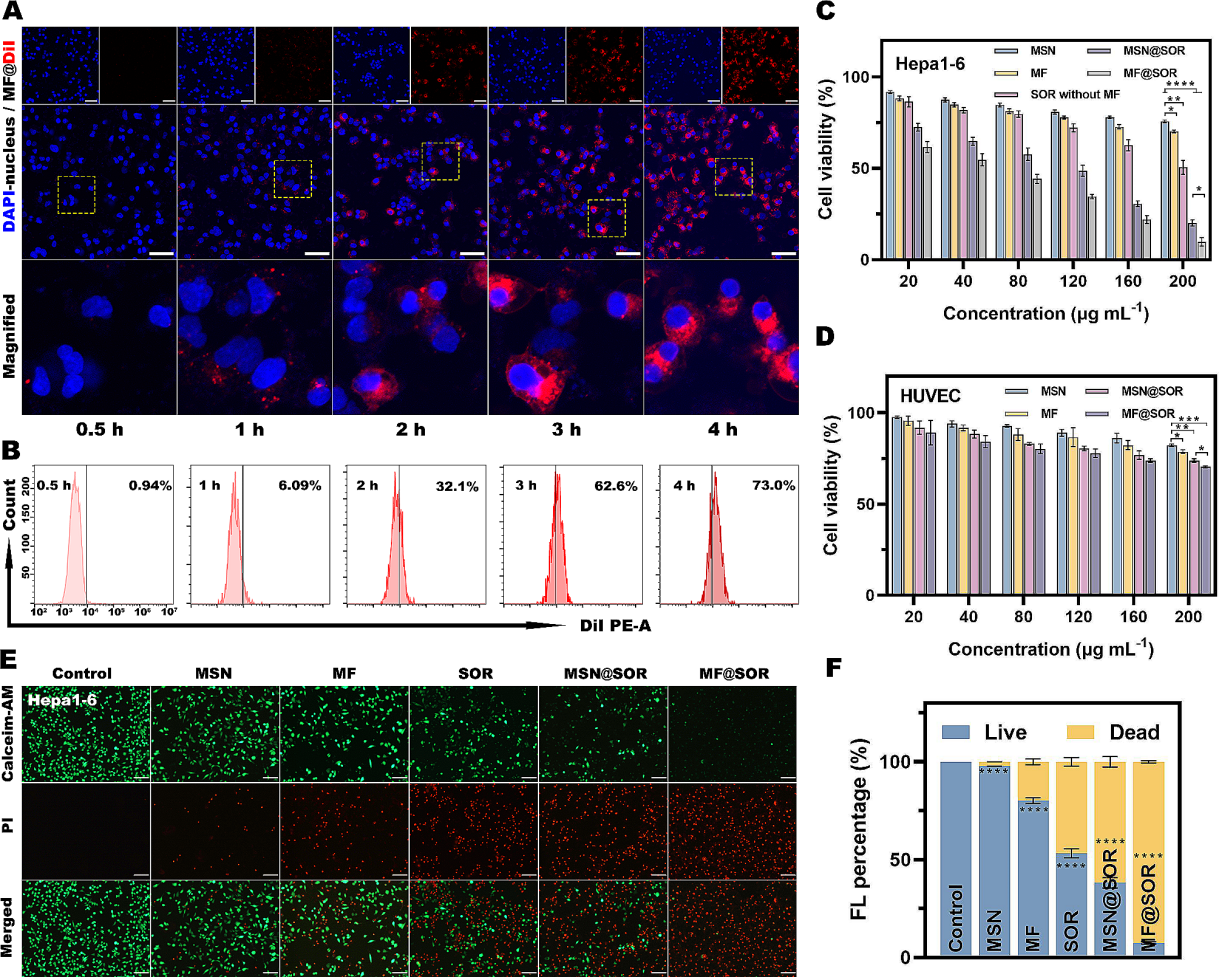
Intracellular endocytosis, cytotoxicity and in vitro antitumor effect of MF@DiI. **(A)** CLSM images of the intracellular endocytosis of MF@DiI (scale bar: 50 μm). **(B)** FCM analysis of the intracellular endocytosis of MF@DiI. Viability of **(C)** Hepa1-6 cells and **(D)** HUVECs after incubation with different concentrations of MSN, MF, MSN@SOR, and MF@SOR for 12 h (*n* = 3). **(E)** CLSM images of live and dead hepa1-6 cells after incubation with different NPs and **(F)** the corresponding FL percentage (scale bars: 100 μm). In panels **(C)** and **(D)**, the data are presented as the mean ± SD, and the *p* values were calculated by ANOVA. ∗ *p* < 0.05, ∗∗ *p* < 0.01, ∗∗∗ *p* < 0.001, ∗∗∗∗ *p* < 0.0001, ns, not significant

In this study, MF@SOR was designed to activate pyroptosis in HCC. Therefore, the uptake of MF@DiI (MF labeled with DiI) by hepa1-6 cells should first be verified. Time-dependent increases in the red fluorescence (FL) signals of MF@DiI around the DAPI-stained nuclei were observed through CLSM, with the maximum intensity observed at 4 h (Fig. 2A). This observation suggested that these tumor cells can robustly uptake MF@DiI. The FCM results revealed an MF@DiI intracellular uptake efficiency of approximately 73% in hepa1-6 cells following a 4-hour incubation with MF@DiI (Fig. 2B), which was in accordance with the CLSM results. These data quantitatively confirmed the ability of hepa1-6 cells to uptake MF@DiI.

#### In vitro cytotoxicity evaluation

The toxicity of the different NPs to hepa1-6 cells and HUVECs was determined by CCK-8 assays (Fig. 2C & D). As the concentration of MSN or MF increased, the viability of hepa1-6 cells decreased. This was attributed to the release of Fe^3+^ and Mn^2+^ in the tumor cells due to the specific intracellular environment, which has a low pH and high GSH content. Comparing MSN and MF at the same concentration, the viability of hepa1-6 cells was lower after SOR loading. This can be attributed to the ability of SOR to activate the noncanonical pathway of pyroptosis and its potential chemotherapeutic effect. In contrast, even in the strongest concentration group for HUVECs, the cellular activity exceeded 70%, indicating the high safety profile of our NPs for normal cells. The IC_50_ value of the MSN@SOR in hepa1-6 cells was calculated to be 79.54 µg mL^− 1^ (for a corresponding SOR concentration of 12.18 µg mL^− 1^), while the IC_50_ value of MF@SOR in hepa1-6 cells was 44.66 µg mL^− 1^ (for a corresponding SOR concentration of 6.84 µg mL^− 1^), both of which are lower than the IC_50_ value of free SOR (26.04 µg mL^− 1^) (Figure S6). Additionally, combining the results of the SOR without MF group, we found that the MF@SOR group exhibits a stronger cytotoxic effect on tumor cells compared to the combination of SOR without MF group and MF group. Therefore, MF and SOR have a synergistic effect in killing tumor cells. In other words, after its incorporation into MSN or MF, a lower concentration of SOR could exert a stronger antitumor effect, this could be attributed to the enhanced internalization of SOR by Hepa1-6 cells facilitated by the NPs. These findings have significant implications for the development of antitumor agents, as they suggest that with the help of nanocarriers, it is possible to achieve a stronger therapeutic effect while reducing the dose of SOR and thus reducing the occurrence of side effects. When the concentration of MF@SOR was increased to 200 µg mL^− 1^, the viability of Hepa1-6 cells decreased to approximately 10%, indicating that the synthesized NPs exhibited a potent cytotoxic effect.

#### In vitro antitumor effects

As shown in Fig. 2E & F, green-stained hepa1-6 cells with good viability were prevalent in the control group, while red-stained apoptotic hepa1-6 cells were almost invisible. In contrast, the number of green-stained cells was reduced after treatment with MSN, MF, SOR, MSN@SOR, or MF@SOR, among which the MF@SOR-treated group displayed the fewest green-stained cells and the most red-stained apoptotic cells. These findings unveiled the efficient antitumor potential of our SOR-loaded bimetallic nanovaccine.

As depicted in Figure S7, at the equivalent treatment concentration, the count of red-stained apoptotic HUVECs was significantly lower than that of hepa1-6 cells in Fig. 2E. This suggested the outstanding safety profile of our nanovaccine on normal cells, in line with the findings from the CCK-8 experiment.

#### Evaluation of pyroptosis and DC maturation in vitro

During pyroptosis, GSDM-N aggregates, causing perforation of the cell membrane and resulting in the release of DAMPs [15] and DNA damage [31]. The presence of free Mn^2+^ in the cytosol can activate cGAS, making it responsive to cytoplasmic dsDNA that is exposed during pyroptosis, thus triggering STING pathway activation and the release of IFN-β [17]. Ultimately, the combined actions of IFN-β and DAMPs promote the maturation of DCs.

To evaluate the severity of ICD, two key DAMPs (CRT, which serves as an ‘eat me’ signal for the phagocytosis of dying cells and participates in stimulating the antigen presentation, and HMGB1, which promotes the stable combination of DCs and dying tumor cell debris, [32] were evaluated using CLSM.

**Fig. 3:**
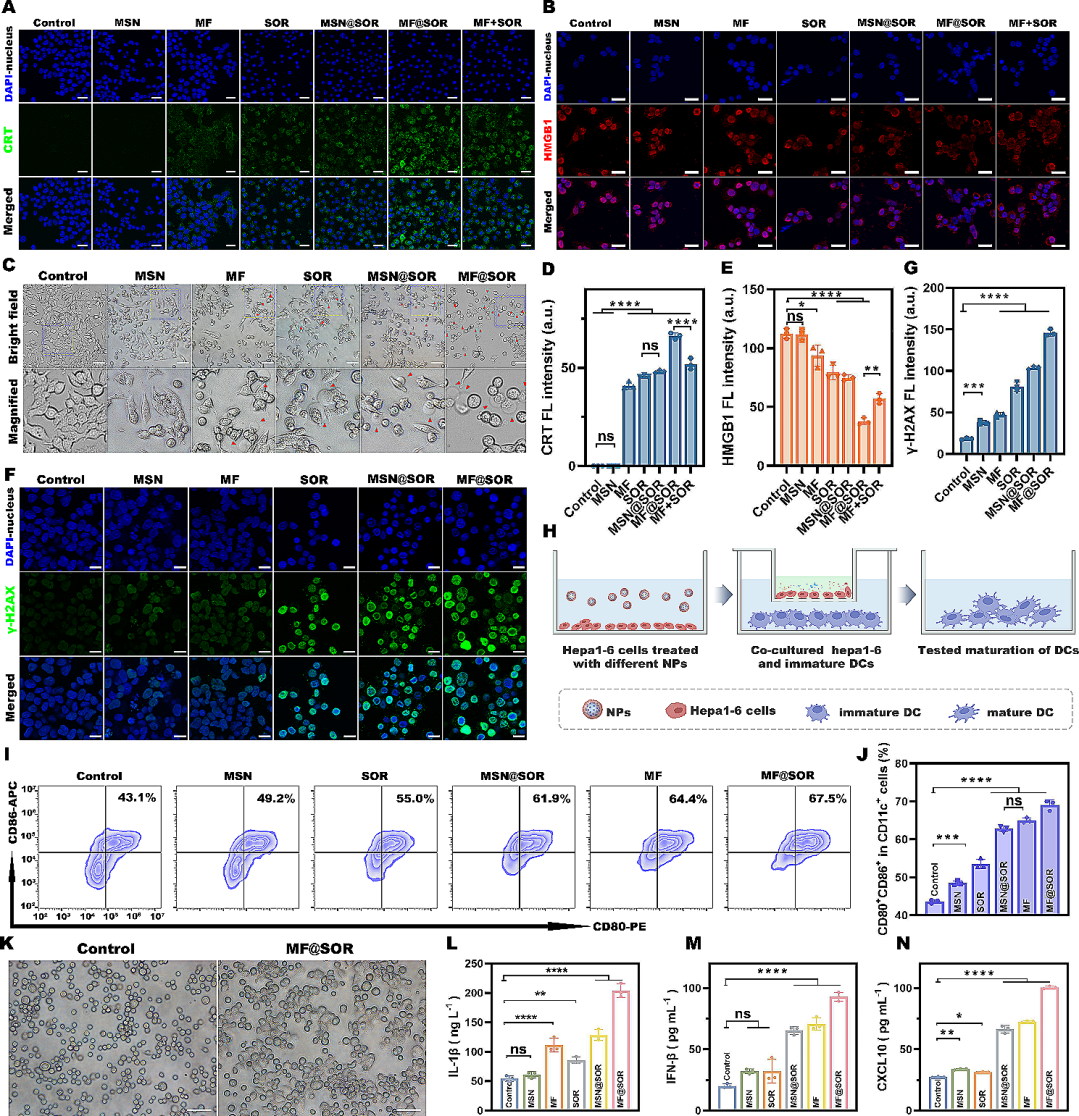
Evaluation of pyroptosis and DC maturation in vitro. **(A)** CLSM images of CRT expression (scale bars: 25 μm). **(B)** CLSM images of HMGB1 expression (scale bars: 15 μm). (**C**) Representative bright field microscopy image of hepa1-6 cells after treatment; the arrows indicate pyroptotic cells. **(D)** Corresponding quantitative analysis of CRT expression (*n* = 3). **(E)** Corresponding quantitative analysis of HMGB1 expression in the nucleus (*n* = 3). **(F)** CLSM images of γ-H2AX staining to visualize DNA fragmentation (scale bars: 25 μm). **(G)** Corresponding quantitative analysis of γ-H2AX expression (*n* = 3). **(H)** Scheme of the coculture system. **(I)** Representative FCM results of mature DCs (CD11c^+^ CD80^+^ CD86^+^) after various treatments. **(J)** Quantitative analysis of mature DCs. **(K)** Representative bright field microscopy image of JAWS II cells after treatment (scale bars: 100 μm). The levels of **(L)** IL-1β, **(M)** IFN-β and **(N)** CXCL10 in the supernatant were measured after various treatments (*n* = 3). In panels **(D)**, **(E)**, **(G)**, **(J)**, **(L)**, **(M)** and **(N)**, the data are presented as the mean ± SD, and the *p* values were calculated by ANOVA. ∗ *p* < 0.05, ∗∗ *p* < 0.01, ∗∗∗ *p* < 0.001, ∗∗∗∗ *p* < 0.0001, ns, not significant

As demonstrated in Fig. 3A & D, hepa1-6 cells were labeled with an anti-CRT antibody. As anticipated, noticeable green FL signals were detected in the MF@SOR-treated group, weaker green FL signals were observed in the MF-, SOR-, MSN@SOR-and MF + SOR- treated groups, negligible green FL signals were observed in the control group and MSN-treated group, indicating the exposure of CRT in the SOR- and MIL-100 (Fe)-treated groups (MF, SOR, MSN@SOR, MF@SOR, MF + SOR), especially the MF@SOR-treated group. This evidence suggested that iron ions and SOR jointly induce ICD. HMGB1 is a nuclear protein, as shown in Fig. 3B & E. The red FL signals in the control and MSN-treated groups were strong and nearly colocalized with the blue-stained cell nuclei. However, in the MF, SOR, MSN@SOR, and MF@SOR treatment groups, the intensity of the nuclear red FL signals gradually diminished, while the red FL signals outside the nucleus gradually increased, especially in the MF@SOR-treated group. Almost all of the red FL signals surrounded the cell nuclei in the MF@SOR-treated group, suggesting that HMGB1 was released from the nuclei into the extracellular environment. Furthermore, in the group co-treated with MF and SOR, we observed stronger red FL signals within the nucleus compared to the MF@SOR group, indicating that iron ions and SOR synergistically promote ICD, rather than simply exhibiting an additive effect.

Bright field images of hepa1-6 cells after different treatments were captured to observe the morphological changes in the cells [33]. In the MF@SOR group, many cells with prominent bubbles (indicated by red arrows) were observed, whereas the hepa1-6 cells in the control group maintained their normal cellular morphology. In addition, in the MSN group, minimal alterations in cell morphology were observed and no signs of swelling were detected, however, in the remaining groups, a few cells exhibited swelling and bubble formation (Fig. 3C). Due to the lack of iron ion modification, the cells swelling in the MSN@SOR group were much less than that in the MF@SOR group. These results indicated that both iron ion- and SOR-induced morphological alterations are typically associated with pyroptosis in cells, suggesting that both are capable of inducing pyroptosis.

Moreover, IL-18 and IL-1β are classic proinflammatory factors that are released during pyroptosis due to destruction of the cell membrane [8, 34]. As shown in Fig. 3L & S8, the levels of both IL-1β and IL-18 increased markedly in the SOR- and MIL-100 (Fe)-treated groups (MF, SOR, MSN@SOR, MF@SOR). The notable observation is that, when compared to MSN@SOR, the presence of Fe in MF@SOR leads to an elevation in IL-1β and IL-18, indicating that SOR and Fe are effective pyroptosis inducers with a synergistic effect.

dsDNA serves as both an indicator of pyroptosis and a crucial trigger for the activation of the STING pathway. Thus, to detect dsDNA that was exposed during pyroptosis, γ-H2AX was used as the detector [16]. IF was used to further detect the formation of γ-H2AX foci, which were visualized as green FL signals (Fig. 3F & G). Compared with the control groups, which presented the lowest green FL, the MF@SOR group presented with more γ-H2AX-positive foci in the cell nuclei. This trend was similar to that of the CRT FL results, suggesting that dsDNA was exposed during pyroptosis. In addition, after different treatments, the levels of the proinflammatory cytokines IFN-β and C-X-C motif chemokine 10 (CXCL10), which are representative indicators of the STING pathway [16], were measured in the cell supernatant of cancer cells. As shown in Fig. 3M & N, the concentrations of IFN-β and CXCL10 in the MF-, MSN@SOR- and MF@SOR-treated groups increased markedly, implying activation of the STING pathway.

The above experiments demonstrated that although MSN@SOR induced pyroptosis and thereby caused the rollover of CRT, the outflow of HMGB1, and the release of proinflammatory cytokines, these represented only single pyroptotic mechanisms induced by SOR, while MF@SOR fully exploited the dual action of Fe and SOR, synergistically enhancing cellular pyroptosis. Additionally, activation of the STING pathway leads to the release of proinflammatory factors, such as IFN-β. These inflammatory substances released by dying tumor cells can be recognized by DCs, which play crucial roles in innate and adaptive immunity. Next, DCs migrate to adjacent draining lymph nodes, where they process antigens into peptides and mature. Finally, mature DCs activate naive T cells, initiating antitumor immune responses [35]. In summary, pyroptosis and activation of the STING pathway can enhance DC maturation and immune system activation.

The maturation of DCs involves increased expression of costimulatory molecules (CD80 and CD86) and the release of proinflammatory cytokines [36, 37]. Therefore, a Transwell system was utilized (Fig. 3H), and the morphology of the cells in the lower chamber was observed under a microscope, afterward, these cells were collected for FCM analysis. Additionally, the culture medium was collected to assess the secretion of the proinflammatory cytokines IL-6 and TNF-α, which are markers of DC maturation. As depicted in Fig. 3K, compared with those in the control group, which were filled with small, uniformly sized cells, the cells in the MF@SOR-treated group were larger and displayed angular shapes, which intuitively reflected DC maturation. As shown in Fig. 3I & J, compared with the control group (43.63% ± 0.39%), the percentages of CD80^+^ CD86^+^ CD11c^+^ DCs in the MSN (48.5% ± 0.51%), SOR (53.53% ± 1.04%), MSN@SOR (62.77% ± 0.63%), MF (64.97% ± 0.6%) and MF@SOR (69.07% ± 1.11%) groups increased sequentially, which was attributed to the pyroptosis-mediated release of proinflammatory substances and activation of the cGAS-STING pathway. Moreover, the concentrations of IL-6 and TNF-α increased markedly (Figure S9). The above findings demonstrate the potent capacity of our nanovaccine to enhance DC maturation, thereby highlighting its superior immunomodulatory potential in tumor treatment.

### Animal experiments

#### Mechanism of pyroptosis and STING pathway activation

**Fig. 4:**
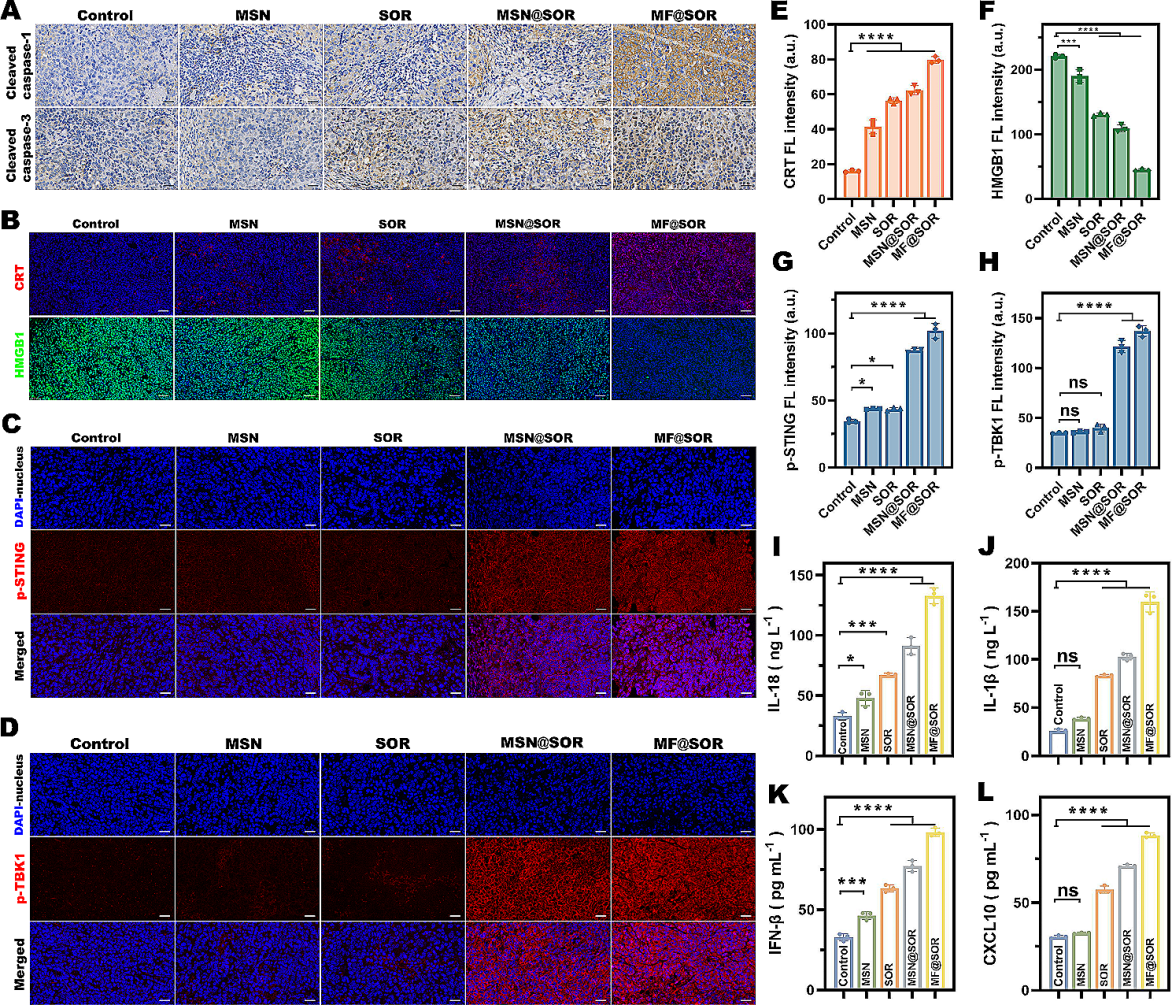
Mechanism of pyroptosis and STING pathway activation. **(A)** IHC of cleaved caspase-1 and cleaved caspase-3 expression in tumor tissues (scale bar: 50 μm). **(B)** IF images of CRT and HMGB1 expression in hepa1-6 tumors after different treatments (scale bar: 50 μm). IF images of **(C)** p-STING and **(D)** p-TBK1 expression in hepa1-6 tumors after different treatments (scale bar: 25 μm). Semiquantitative FL intensity analysis of **(E)** CRT, **(F)** HMGB1, **(G)** p-STING and **(H)** p-TBK1 staining (*n* = 3). The levels of **(I)** IL-18, **(J)** IL-1β, **(K)** IFN-β and **(L)** CXCL10 in mouse serum after various treatments (*n* = 3). In panels **(E)**, **(F)**, **(G)**, **(H)**, **(I)**, **(J)**, **(K)** and **(L)**, the data are presented as the mean ± SD. All *p* values were calculated by ANOVA. ∗ *p* < 0.05, ∗∗ *p* < 0.01, ∗∗∗ *p* < 0.001, ∗∗∗∗ *p* < 0.0001, ns, not significant

Chemotherapy-induced pyroptosis is mediated by the nonclassical caspase-3-related pathway, [13, 14] while Fe-induced pyroptosis was recently reported to be altered by the canonical caspase-1-related pathway [10–12]. Cleaved caspase-3 cleaves GSDME and leads to the release of the GSDME-N domain, while cleaved caspase-1 cleaves GSDMD, which results in the release of the GSDMD-N domain [9]. To study the mechanism by which the nanovaccine caused pyroptosis, we used IHC to detect cleaved caspase-1 and cleaved caspase-3 in tumor tissues following various treatments. As shown in Fig. 4A & S10, the expression of cleaved caspase-1 was significantly increased in the MIL-100 (Fe)-treated group (MF@SOR), while cleaved caspase-3 expression was noticeably increased in all of the SOR-treated groups (SOR, MSN@SOR, and MF@SOR). These results indicated that MF@SOR induced tumor cell pyroptosis through both classical and nonclassical pathways. GSDME-N and GSDMD-N migrate to the cellular membrane and assemble membrane pores, inducing cell swelling, membrane rupture, and the release of DAMPs, leading to the activation of immune responses associated with pyroptosis [7]. As shown by the IF results, the CRT-related red FL signals exhibited progressive increases in the following order: the control, MSN, SOR, MSN@SOR and MF@SOR groups. Concurrently, the green FL signals indicating HMGB1 within the cell nuclei decreased in the same order (Fig. 4B, E & F). These results were consistent with those obtained from the cell experiments. The concentrations of IL-18 and IL-1β in mouse serum showed increasing trends in the order of the control, MSN, SOR, MSN@SOR and MF@SOR groups, which also indicated the occurrence of pyroptosis (Fig. 4I & J).

The phosphorylation of STING, TBK1, and IRF3 is a hallmark of cGAS-STING activation [38]. Thus, IF staining of tumor tissues for p-STING (red) (Fig. 4C & G), p-TBK1 (red) (Fig. 4D & H) and p-IRF3 (red) (Figure S11) was performed after different treatments to validate our hypothesis. The results showed that the red FL signal intensity in the tumor tissue sections from mice treated with MSN@SOR or MF@SOR was greater than that in the other groups, indicating that the increased expression of cGAS-STING-related proteins in tumor tissues could be attributed to the simultaneous presence of dsDNA and manganese ions. Furthermore, the concentrations of IFN-β and CXCL10 in mouse serum were greater in the MSN@SOR and MF@SOR groups than in the other groups, as measured by ELISA, also suggesting activation of the cGAS-STING pathway (Fig. 4K & L).

In summary, through the aforementioned experiments, we validated the antitumor mechanisms of MF@SOR. Specifically, these nanovaccines elicit a potent pyroptotic response, which subsequently triggers activation of the cGAS-STING pathway via the recognition of dsDNA generated during pyroptosis. This intricate interplay between pyroptosis and cGAS-STING activation culminates in the efficient elimination of malignant cells.

#### In vivo immunomodulation

In our study, we aimed to explore and evaluate the concerted immunostimulatory effects elicited by combined pyroptosis and STING activation. On the basis of our above findings from cellular experiments showing that our nanovaccines promoted the maturation of DCs, we further examined the immunostimulatory impacts of the different NPs in vivo by collecting blood, tumor, spleen, and TDLNs tissues from mice treated with various NPs to assess the relevant immune markers.

**Fig. 5:**
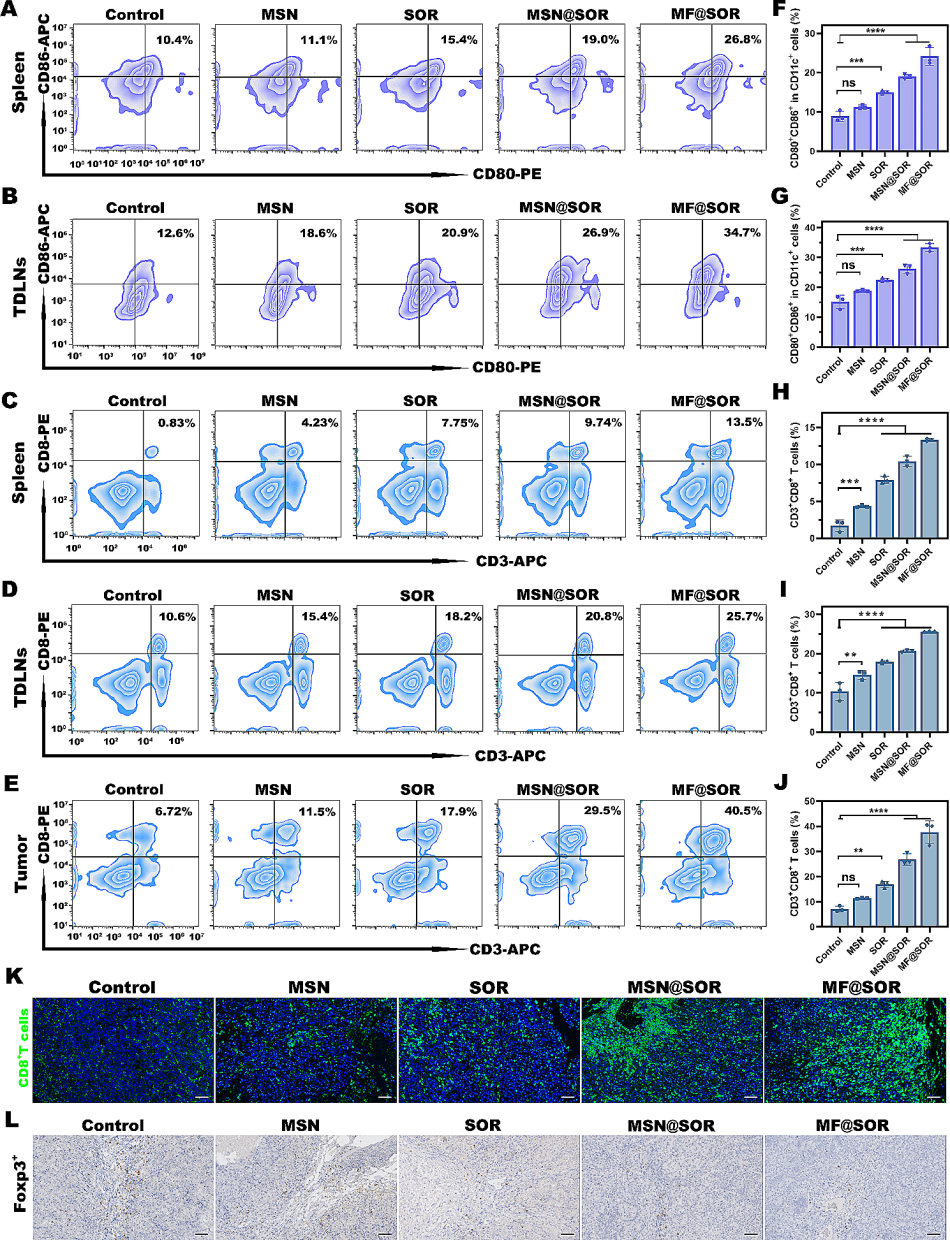
Analysis of immunomodulation in the primary tumor model. DC maturation (CD11c^+^ CD80^+^ CD86^+^) in the **(A)** spleens and **(B)** TDLNs. Quantitative analysis of DC maturation (CD11c^+^ CD80^+^ CD86^+^) in the **(F)** spleens and **(G)** TDLNs (*n* = 3). FCM analysis of CD3^+^CD8^+^ T cells in the **(C)** spleens, **(D)** TDLNs, and **(E)** tumors, and (**H**, **I**, and **J**) quantitative analysis (*n* = 3). **(K)** IF images of CD8^+^ T cells in hepa1-6 tumors after different treatments (scale bar: 50 μm). **(L)** IHC of Foxp3^+^ expression in tumor tissues (scale bar: 50 μm). In panels **(F)**, **(G)**, **(H)**, **(I)** and **(J)**, the data are presented as the mean ± SD. All *p* values were calculated by ANOVA. ∗ *p* < 0.05, ∗∗ *p* < 0.01, ∗∗∗ *p* < 0.001, ∗∗∗∗ *p* < 0.0001, ns, not significant

The FCM results indicated that the proportion of mature DCs in the spleen of mice treated with MF@SOR NPs was approximately 24.17% ± 1.88%, which was more than twice as high as that in the control group (8.9% ± 1.08%) (Fig. 5A & F). Moreover, the proportions of mature DCs (CD80^+^ CD86^+^ CD11c^+^) in the TDLNs of mice treated with different NPs were 18.8% ± 0.28% (MSN), 22.47% ± 0.46% (SOR), 26.23% ± 1.24% (MSN@SOR), and 33.33% ± 1.1% (MF@SOR) (Fig. 5B & G). The proportion of mature DCs in each of the treatment groups was higher than that in the control group (15.13% ± 1.82%). In addition, the measurements of IL-6 (Figure S12A) and TNF-α (Figure S12B) contents in mouse serum using ELISAs showed a comparable trend to the FCM results, again suggesting DC maturation. In summary, the aforementioned investigations provided compelling evidence to support the potent capability of the synthesized nanovaccines to facilitate the maturation of DCs, thereby establishing a solid groundwork for the subsequent generation of CTLs.

Given that mature DCs can activate T lymphocytes and elicit effective adaptive immune responses, we investigated the distribution of CD3^+^ CD8^+^ T cells in tumor tissues, TDLNs, and spleens of mice following various treatments. Compared to those in the control group, the MF@SOR group exhibited a significant increase in the occurrence rate of CD3^+^ CD8^+^ T cells in spleens (Fig. 5C & H), TDLNs (Fig. 5D & I), and tumors (Fig. 5E & J), followed by the MSN@SOR group, the SOR group, and the MSN group, suggesting an enhanced systemic immune response. These findings indicated that MSN, SOR, MSN@SOR or MF@SOR induced systematic immune responses to varying degrees. In addition, the IF results of the CD8^+^ T cell content in tumors was consistent with the FCM data (Fig. 5K & S13A). Abundant studies have reported that tumor cells undergoing pyroptosis can release proinflammatory cytokines (IL-1β) which can inhibit Tregs, [8] thus, immunosuppressive Tregs within the tumor were analyzed using IHC. The findings demonstrated that MF@SOR significantly reduced the number of Foxp3^+^ cells, indicating relief from immunosuppression (Fig. 5L & S13B).

Overall, the above results demonstrated the significant potential of MF@SOR to stimulate antitumor immune responses and alleviate immunosuppression. These findings elucidated the synergistic immune-enhancing effects of pyroptosis and cGAS-STING pathway activation. This potent immune response may play a crucial role in extending the survival rate of mice.

#### In vivo antitumor effect

**Fig. 6:**
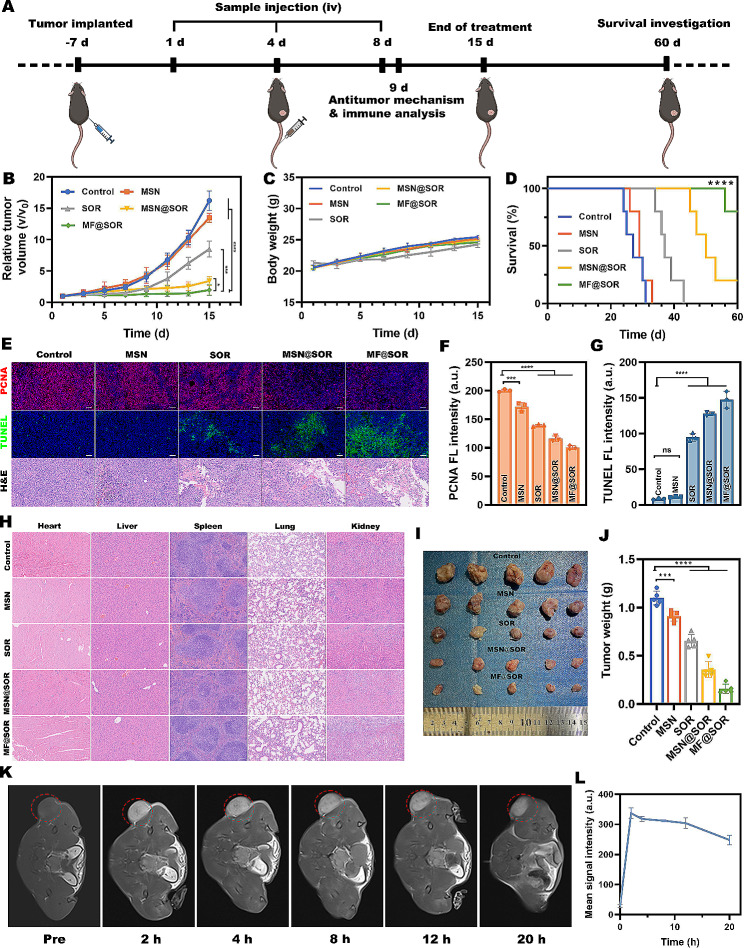
In vivo antitumor effect and MRI. **(A)** Schematic illustration of the experimental design of the primary tumor models (created with BioRender.com). **(B)** Time-dependent relative tumor volume growth curves in the different groups (*n* = 5). **(C)** Time-dependent body weight curves of mice in different groups (*n* = 5). **(D)** Survival curves of mice in different groups (*n* = 5). (E) Representative images of TUNEL, PCNA and H&E analysis in each group (scale bar: 50 μm). Semiquantitative FL intensity analysis of **(F)** PCNA and **(G)** TUNEL staining (*n* = 3). **(H)** H&E staining of the major organs in each group after various treatments (scale bar: 100 μm). Representative **(I)** digital photos and **(J)** weights of dissected tumors from different groups at the end of treatment (*n* = 5). **(K)** T1 MRI of subcutaneous models at different time points. **(L)** Corresponding change mean signal intensity in the tumor regions in the subcutaneous models at different time intervals (*n* = 3). In panels **(B)**, **(C)**, **(D)**, **(F)**, **(G)**, **(J)** and **(L)**, the data are presented as the mean ± SD. All *p* values were calculated by ANOVA. ∗ *p* < 0.05, ∗∗ *p* < 0.01, ∗∗∗ *p* < 0.001, ∗∗∗∗ *p* < 0.0001, ns, not significant

Figure 6 A illustrates the protocol by which C57BL/6J Hepa1-6 tumor-bearing mice were treated. None of the experimental groups exhibited any abnormalities in body weight (Fig. 6C). In comparison to the control group that received saline treatment, tumor growth was slightly suppressed in the MSN treatment group. Furthermore, the MSN@SOR group exhibited significant tumor inhibition but remained inferior to that of the MF@SOR group, due to the absence of iron ion-mediated activation of the classical pyroptosis pathway (Fig. 6B & S14). Next, the tumor weight results and representative images of dissected tumors obtained 14 days after treatment further provided visual evidence of the considerable efficacy of MF@SOR in inhibiting tumor growth (Fig. 6I & J). This observation aligned with the outcome observed in terms of the relative alteration of tumor volume, providing unequivocal validation for the synergy of MSN in combination with SOR and MIL-100 (Fe), which ultimately resulted in potentiated tumoricidal efficacy. Then, PCNA, TUNEL, and H&E staining assays were conducted to assess the efficacy of the treatment in inhibiting tumor growth (Fig. 6E, F & G). The PCNA images exhibited faint FL signals associated with proliferation in the two groups administered with MSN@SOR or MF@SOR, while the intensities of the proliferation-related FL signals were slightly higher in the SOR-treated group and significantly higher in the control group and MSN-treated group. On the contrary, the TUNEL images showed strong apoptosis-related FL signals in the MSN@SOR and MF@SOR groups, weaker FL signals in the SOR-treated group and almost negligible FL signals in both the control group and MSN-treated group. As shown by the H&E staining images, abundant apoptosis and necrosis were observed in the MSN@SOR- and MF@SOR-treated groups. In the groups treated with SOR and MSN, the areas of necrosis and apoptosis decreased gradually, and almost no necrosis or apoptosis was observed in the control group. These results were consistent with those obtained from PCNA and TUNEL staining, which together indicated that our nanovaccine exhibited potent potential to suppress tumor proliferation.

In addition, the survival times of the mice were recorded to evaluate whether MF@SOR treatment could extend their survival rate. The survival times were significantly extended in all groups receiving treatment compared to the control group, especially in the MF@SOR-treated group. As shown in Fig. 6D, by the 60th day, all mice in the control, MSN, and SOR groups had died, with only one mouse surviving in the MSN@SOR group, while the MF@SOR group still had four surviving mice. Compared to the MSN@SOR group, the survival time of the MF@SOR group was significantly increased, which relied on the synergistic pyroptosis of Fe and SOR. Furthermore, examination of H&E-stained images of major organs dissected from mice after 14 days of treatment revealed no evident pathological damage or inflammatory lesions (Fig. 6H), indicating minimal adverse effects during the course of tumor treatment. These results demonstrated the potent antitumor efficacy of our nanovaccines, which led to a significant extension in lifespan.

#### In vivo MRI

Having established the imaging and TME responsiveness of the nanovaccine through prior in vitro experiments, we subsequently evaluated the T1 MRI properties of the MF@SOR in vivo. The T1 MRI contrast signal reached its maximum 2 h after administration, indicating the swift accumulation of the NPs at the tumor site through the EPR effect (Fig. 6K & L). These findings indicate that the nanovaccine possessed good T1 MRI properties. In the grayscale images, we also observed that after injection, the T1 MRI contrast signal exhibited a notable increase in the tumor region compared to the surrounding tissue, highlighting the ability of the nanovaccine to respond to imaging in the TME, which is characterized by a low pH and high GSH content. In addition, through in vivo dynamic MRI, it was discerned that the tumor region displayed a gradual and protracted decrease in the T1 MRI contrast signal. Notably, even after a span of 20 h, only slight attenuation was observed. This compelling evidence underscores the prolonged retention of our nanovaccine within the tumor milieu, thereby substantiating its potential for sustained immune stimulation within the tumor region.

#### Long-term immune effects in the recurrent and metastasis models

**Fig. 7:**
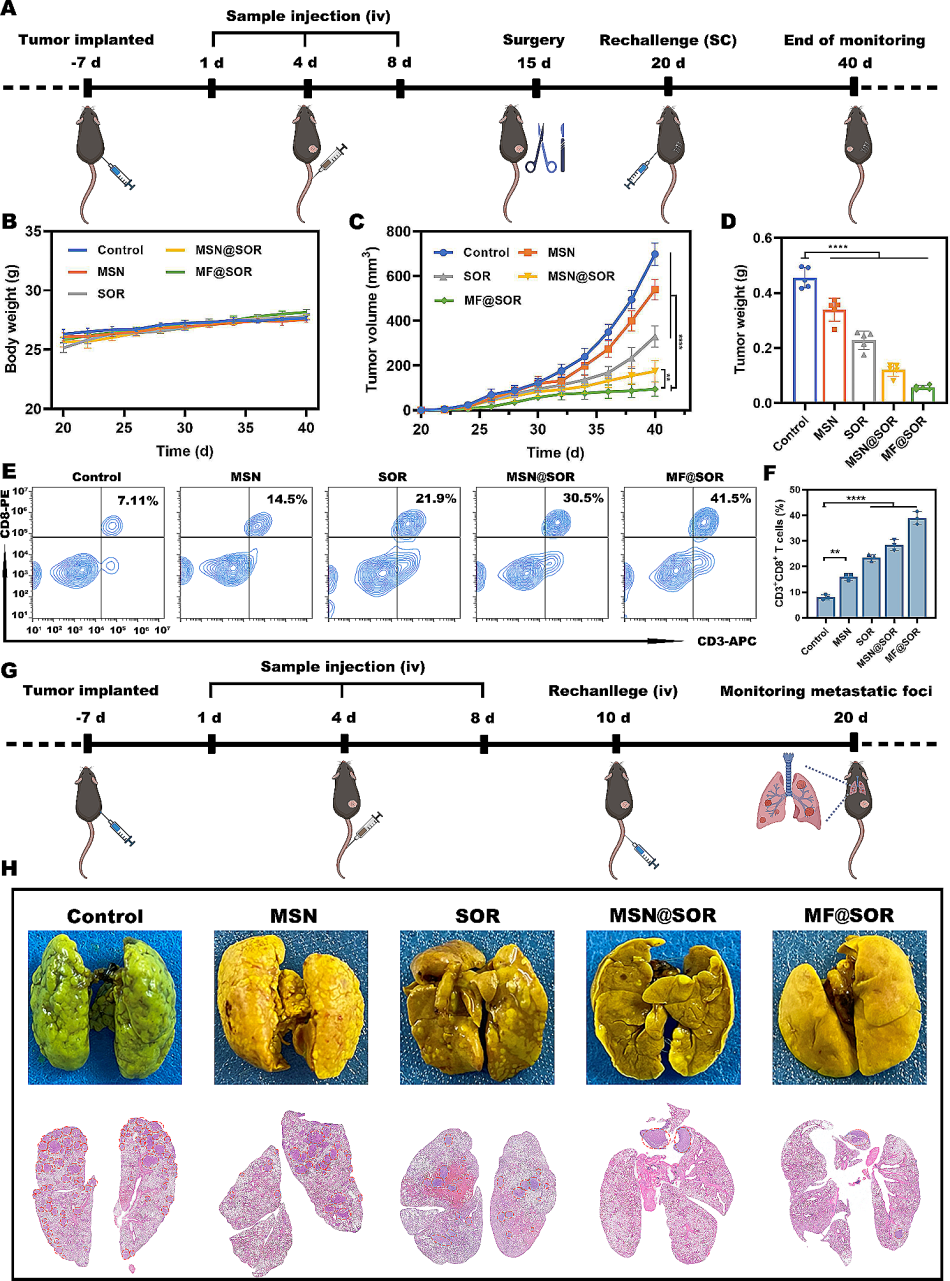
Long-term immune effects in the recurrent and metastatic models. **(A)** Schematic illustration of the animal experimental design for the recurrent tumor model (created with BioRender.com). **(B)** Body weight changes in recurrent tumor-bearing mice (*n* = 3). **(C)** Recurrent relative tumor volume growth curves for each group (*n* = 3). **(D)** Weights of the excised recurrent tumors in each group (*n* = 3). **(E)** FCM analysis of CD3^+^CD8^+^ T cells in the recurrent tumors in each group and **(F)** quantitative analysis. **(G)** Schematic illustration of the animal experimental design for the lung metastasis tumor model (created with BioRender.com). **(H)** Representative photographs and H&E staining images of lung tissue from mice. In panels **(B)**, **(C)** and **(D)**, the data are presented as the mean ± SD. All *p* values were calculated by ANOVA. ∗ *p* < 0.05, ∗∗ *p* < 0.01, ∗∗∗ *p* < 0.001, ∗∗∗∗ *p* < 0.0001, ns, not significant

After observing the notable immunostimulation and remarkable tumor inhibition effect of the nanovaccines in primary liver cancer mouse models, the persistent immune memory was also examined in tumor recurrence and metastasis models (Fig. 7A & G). No notable abnormalities were observed in the body weight of mice (Fig. 7B). As we speculated, compared to all the other groups, the mice that received MF@SOR treatment showed a significant decrease in the size (Fig. 7C, S15 & S16) and weight (Fig. 7D) of the recurrent tumors, while the incidence of lung metastasis in the MF@SOR-treated group was also significantly reduced (Fig. 7H & S17). These data indicated that our nanovaccines might generate effective antitumor immune memory, leading to sustained protection against tumor recurrence and metastasis. Next, we used FCM to measure the percentages of CD3^+^ CD8^+^ T cells, which play a crucial role in killing cancer cells, as the main effector immune cells, in the rechallenged tumor models. FCM analysis revealed that CD3^+^ CD8^+^ T cells exhibited the most significant increase in expression in mice pretreated with MF@SOR (38.97% ± 2%), followed by the mice receiving MSN@SOR (28.43% ± 1.72%), SOR (23.37% ± 1.09%), MSN (16.03% ± 1.08%) and control treatment (8.03% ± 0.81%) (Fig. 7E & F). Moreover, we verified the ability of MF@SOR to promote the transformation of splenic CD8^+^ T cells into central memory T cells (CD3^+^ CD8^+^ CD44^+^ CD62L^−^). The proportion of central memory T cells in the spleens of mice treated with MF@SOR was the highest (28.4% ± 2.17%), followed by the mice receiving MSN@SOR (20.8% ± 1.5%), SOR (14.63% ± 0.21%), MSN (10.47% ± 0.21%) and control treatment (8.3% ± 0.35%) (Figure S18), which was in line with the previously mentioned results obtained from the primary tumor models. These findings suggested that the MF@SOR could generate long-lasting immune memory and stimulate robust immune reactions to tumor recurrence and metastasis.

In summary, the activation of two pyroptosis pathways and the cGAS-STING pathway by MF@SOR not only effectively triggered a systemic antitumor immune response but also promoted long-term immune memory protection.

#### Tumor accumulation, biodistribution, and clearance rates of MF@SOR

**Fig. 8:**
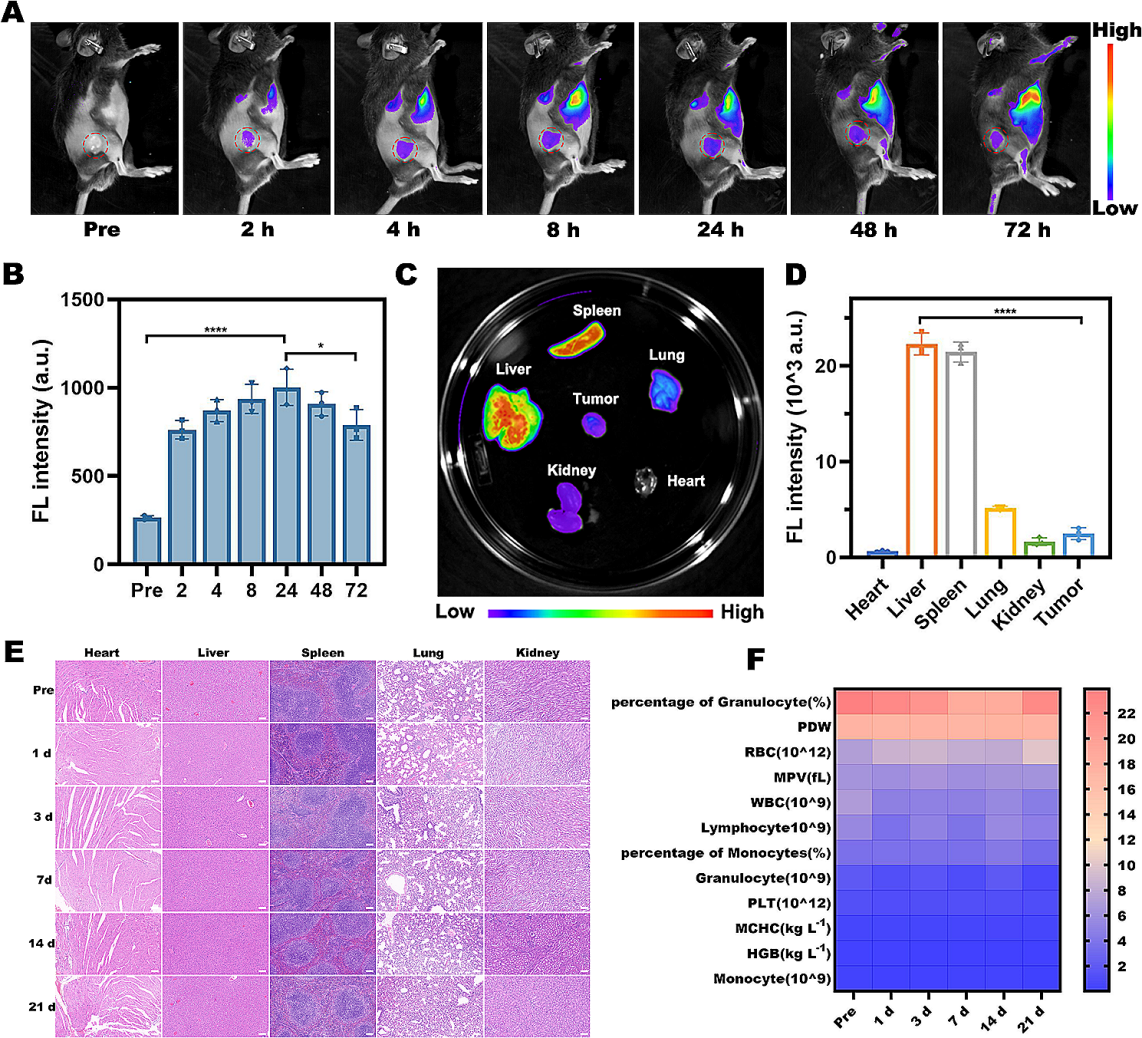
In vivo FLI and biosafety assessment. **(A)** In vivo FL images of Hepa1-6 tumor-bearing mice. **(B)** Alterations in FL signal intensity within the tumor regions at the respective time intervals (*n* = 3). **(C)** FL images of the major organs and tumors removed from mice 72 h postinjection of MF@DiR and **(D)** corresponding quantitative analysis (*n* = 3). **(E)** H&E staining images of the major organs (scale bar: 200 μm). **(F)** Routine blood examination results from Kunming mice intravenously injected with the MF@SOR at different time points. In panels **(B)** and **(D)**, the data are presented as the mean ± SD. All *p* values were calculated by ANOVA. ∗ *p* < 0.05, ∗∗ *p* < 0.01, ∗∗∗ *p* < 0.001, ∗∗∗∗ *p* < 0.0001, ns, not significant

In vivo visualization of the biodistribution and tumor accumulation of the nanovaccine was achieved using FLI and ICP-MS. The FL intensity at the tumor site reached a clear peak 24 h after MF@DiR (MF labeled with DiR) administration and then gradually decayed until 72 h, as shown in Fig. 8A. This was consistent with the quantitative assessment of the FL signal intensity (Fig. 8B) and ICP-MS (Figure S19). These results indicated that MF@DiR were able to effectively accumulate at the tumor site through the EPR effect and were then gradually eliminated by the body. Subsequently, ex vivo FLI of major organs and extracted tumors was performed (Fig. 8C). The images, quantitative analysis and ICP-MS revealed that the liver exhibited the most intense FL intensity with the highest FL signal, followed by the spleen, lung, tumor, kidney and heart (Fig. 8D & S20), revealing the biodistribution of the NPs. The liver showed the highest accumulation of NPs, which could be attributed to its rich blood perfusion and phagocytosis of foreign substances by Kupffer cells [39]. Moreover, we measured the blood Mn concentrations at different time points, the results showed the clearance rates of MF@SOR in the blood (Figure S21).

#### Biosafety of the MF@SOR

Ensuring the biosafety of nanomaterials is crucial for their clinical translation. We therefore conducted analyses of routine blood and blood biochemical indexes in Kunming mice at different time points (0, 1, 3, 7, 14, and 21 days) after administering the MF@SOR to assess the acute (1 day), short-term (3 and 7 days), and long-term (14 and 21 days) biosafety of the NPs (Fig. 8F & S22). The results revealed no significant abnormalities within the group receiving treatment in comparison to the control group, indicating no obvious systematic toxicity. Furthermore, H&E staining was used to histopathologically examine the major organs extracted from the mice at various time points, and the outcomes did not significantly differ between the group that received intravenous injection of MF@SOR and the control group (Fig. 8E). These findings highlight the excellent in vivo biocompatibility of the NPs and demonstrate the great potential of MF@SOR for future clinical translation.

## Conclusion

In summary, we successfully synthesized bimetallic nanovaccines that exhibit smart responsiveness to specific chemical signals in the TME, thereby enabling the synchronous delivery of multiple components, imaging monitoring and the activation of pyroptosis and the cGAS-STING pathway, which synergistically promote DC maturation. Consequently, there was a reduction in the number of Tregs within the tumor region, alleviation of immunosuppression, and significant recruitment of CD8^+^ T cells to the tumor area. This led to the elimination of the primary tumor while generating long-lasting antitumor immune memory, effectively inhibiting tumor recurrence and metastasis and prolonging the survival time of mice. Overall, MF@SOR is a safe and effective nanovaccine, and this is an innovative strategy for the management of HCC via the utilization of metalloimmunotherapy.

In forthcoming investigations, we will elucidate the prospective capacity of nanovaccines to augment the recruitment of diverse immune cell populations, including but not limited to natural killer (NK) cells. Additionally, we will continue to refine our material to enhance its tumor-targeting capabilities.

### Electronic supplementary material

Below is the link to the electronic supplementary material.

Supplementary Material 1

## Data Availability

No datasets were generated or analysed during the current study.
