# Supplementary material for: Smart responsive Fe/Mn nanovaccine triggers liver cancer immunotherapy via pyroptosis and pyroptosis-boosted cGAS-STING activation
Source: J Nanobiotechnology. 2024 Mar 6;22:95. doi: 10.1186/s12951-024-02354-2 (PMC10918897; doi:10.1186/s12951-024-02354-2)
Supplement: Supplementary file 1 — Supplementary Material 1 [file 12951_2024_2354_MOESM1_ESM.docx]

**Smart Responsive Fe/Mn Nanovaccine Triggers Liver Cancer Immunotherapy via Pyroptosis and Pyroptosis-Boosted cGAS-STING Activation**

*Qianying Du^1,2^, Ying Luo^1,2^, Lian Xu^1,2^, Chier Du^2^, Wenli Zhang^1,2^, Jie Xu^1^, Yun Liu^1^, Bo Liu^1^, Sijin Chen^1^, Yi Wang^1^, Zhigang Wang^2^, Haitao Ran^2^, Junrui Wang^1,2*^, and Dajing Guo^1*^*

1. Department of Radiology, Second Affiliated Hospital of Chongqing Medical University, Chongqing 400010, P. R. China
2. Chongqing Key Laboratory of Ultrasound Molecular Imaging & Department of Ultrasound, Second Affiliated Hospital of Chongqing Medical University, Chongqing 400010, P. R. China

Correspondence: guodaj@163.com (Dajing Guo), drwangjunrui@163.com (Junrui Wang).

**Supplementary equations**

**Equation 1.** Encapsulation efficiency (EE) (%) = (mass of SOR in NPs) / (total initial mass of SOR) × 100%

**Equation 2.** Loading capacity (LC) (% w/w) = (mass of SOR in NPs) / (total mass of SOR, MSN and MIL-100(Fe)) × 100%

**3. Supplementary figures**


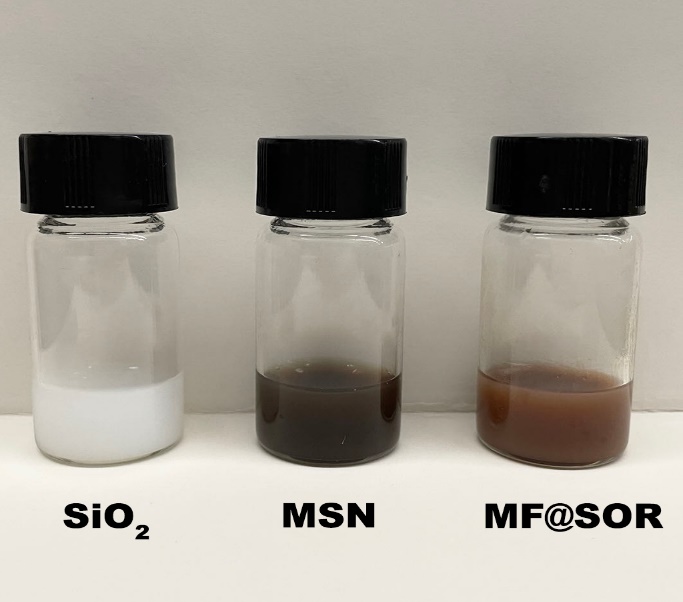


**Figure S1.** Representative photographs of different NPs


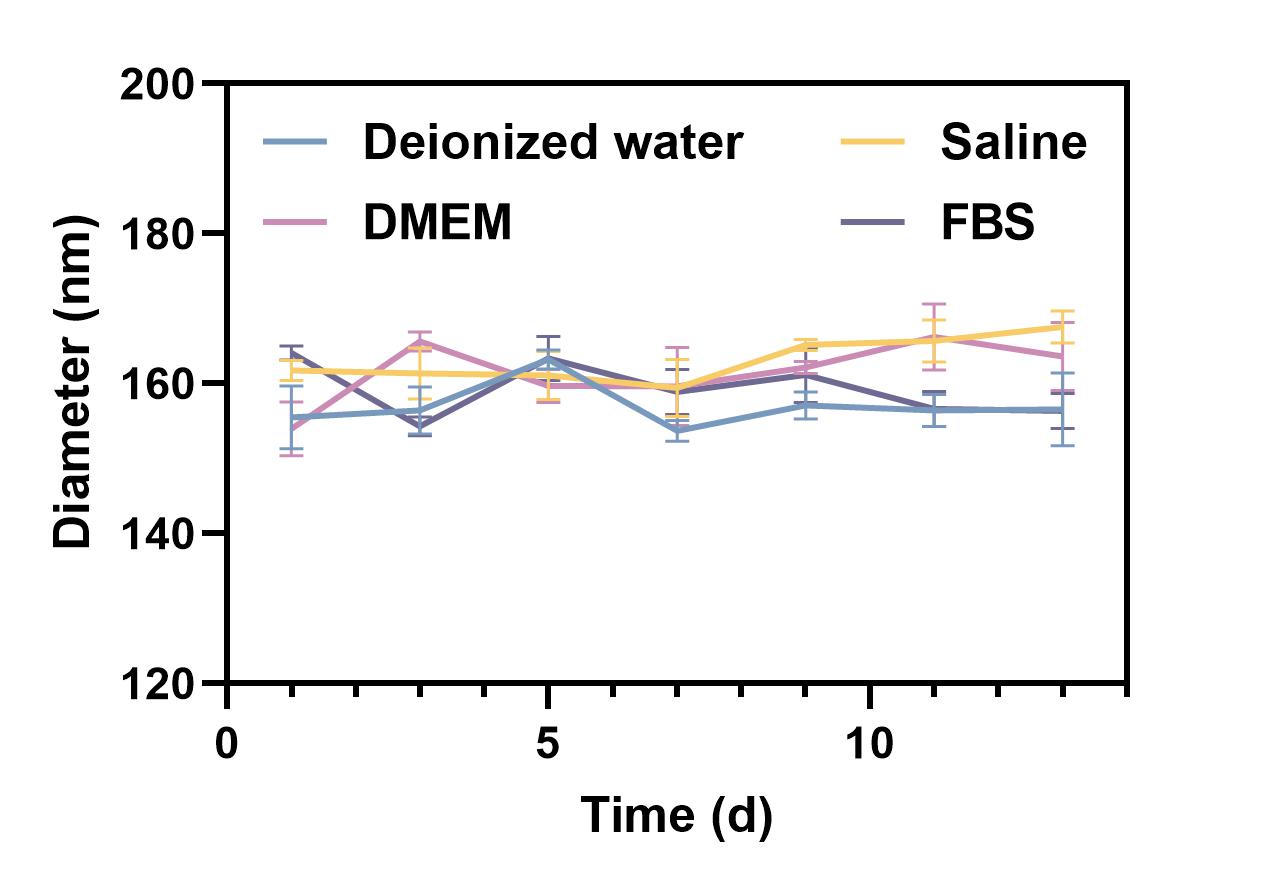


**Figure S2**. The colloidal stability of the MF@SOR in deionized water, saline, DMEM and FBS. Data are represented as mean ± SD (n = 3).


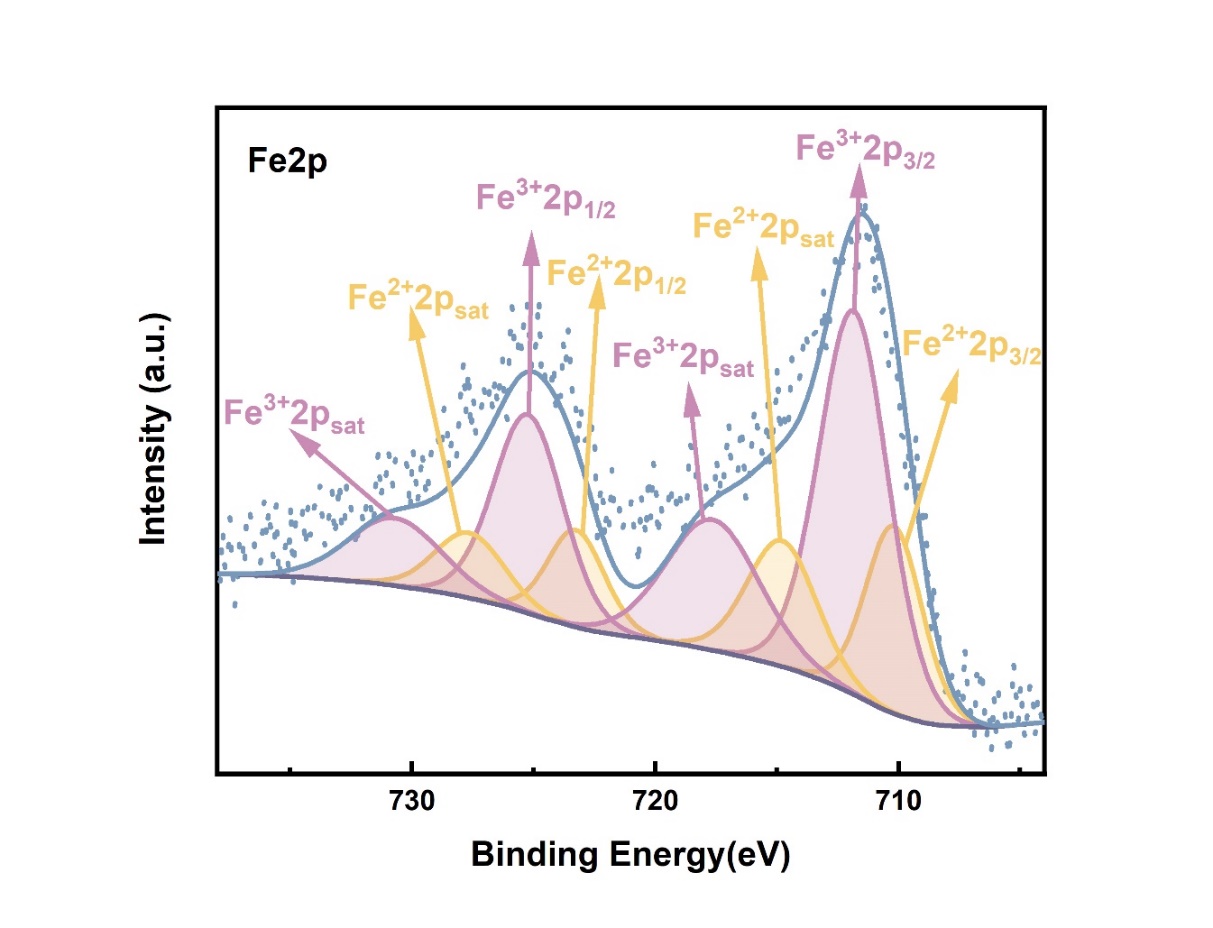


**Figure S3**. XPS of Fe in MF@SOR.

**
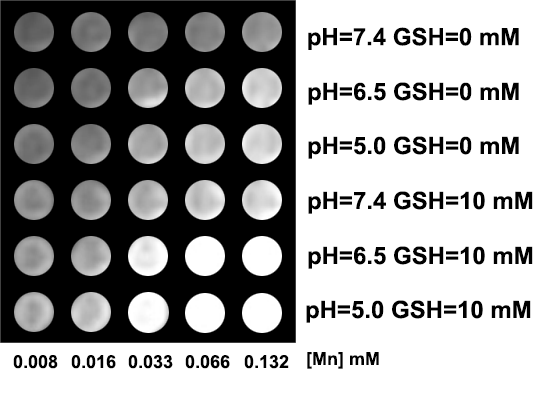
**

**Figure S4**. T1-weighted MRI images of the nanovaccines in various environments at 3.0 T MRI scanner.


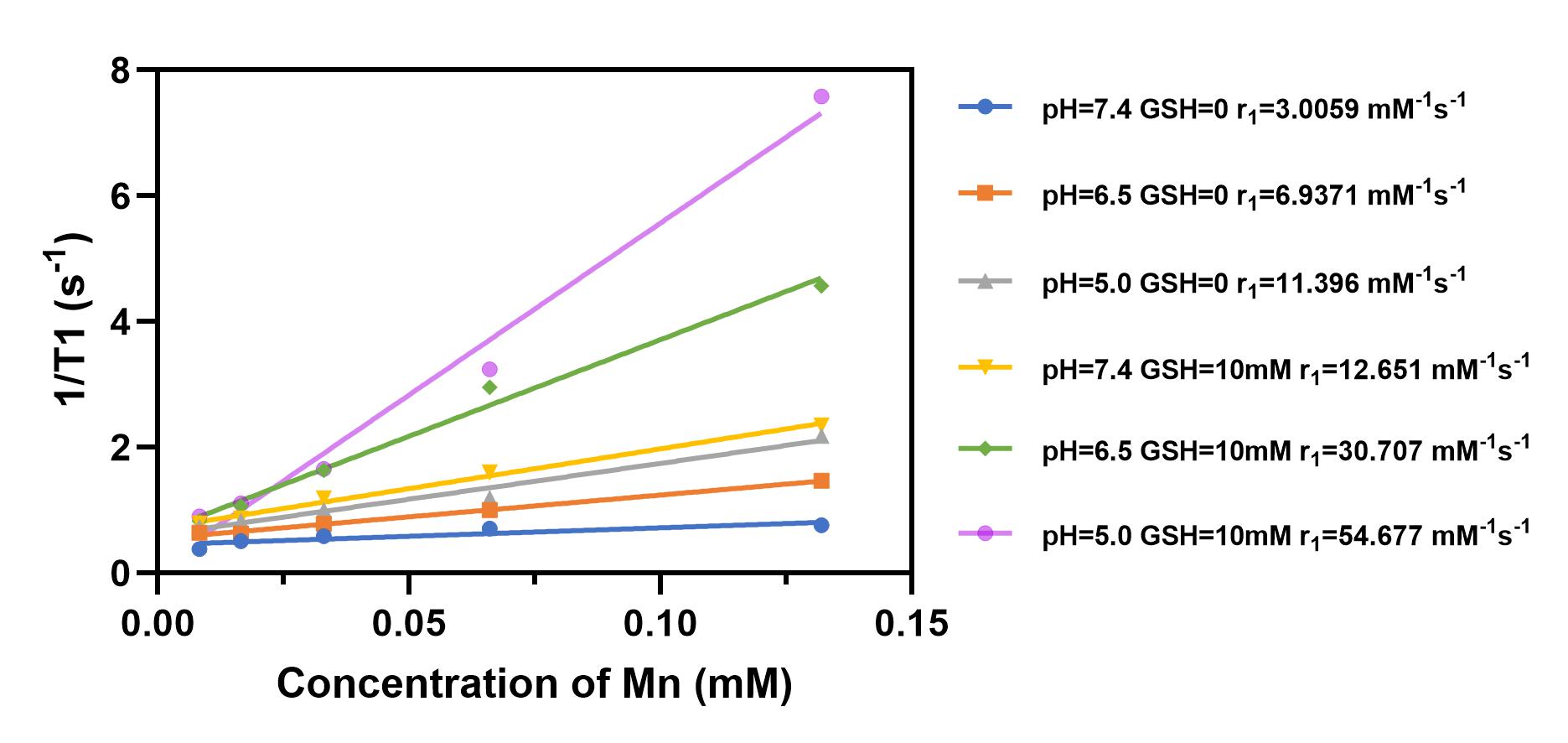


**Figure S5**. The r_1_ values of the nanovaccines in different environments at 3.0 T MRI scanner.


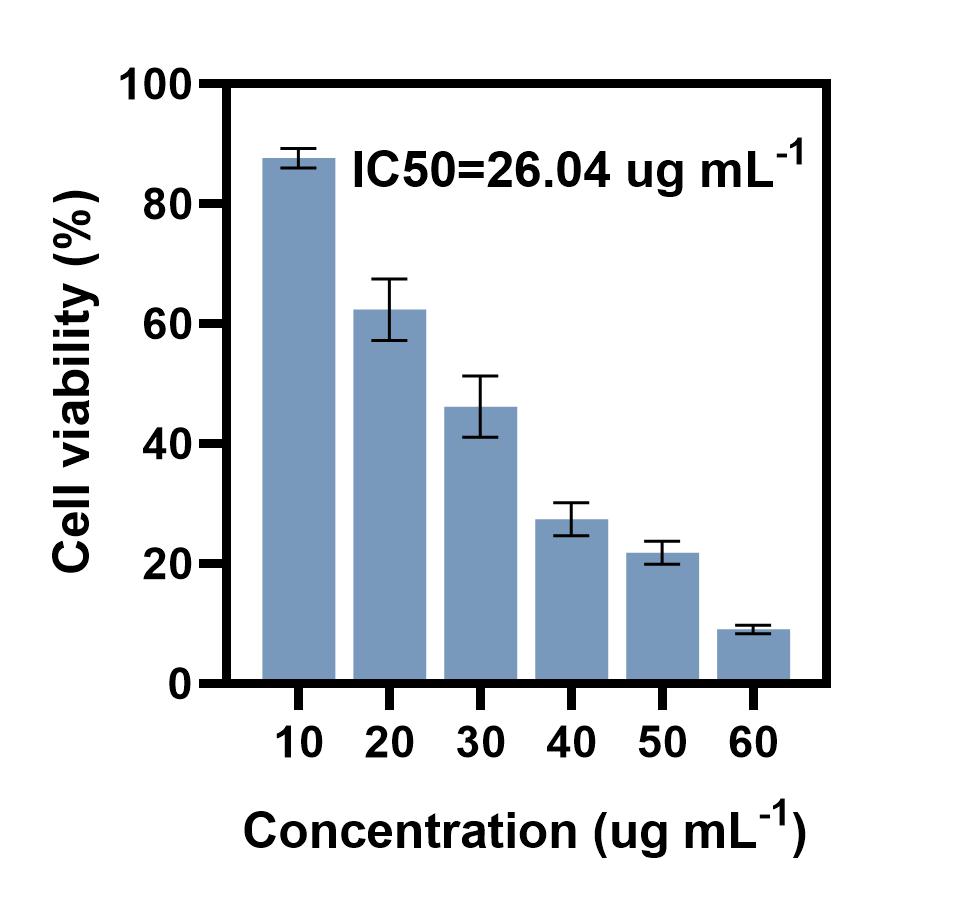


**Figure S6**. Cytotoxicity of SOR on Hepa1-6 cells. Data are represented as mean ± SD (n = 3).


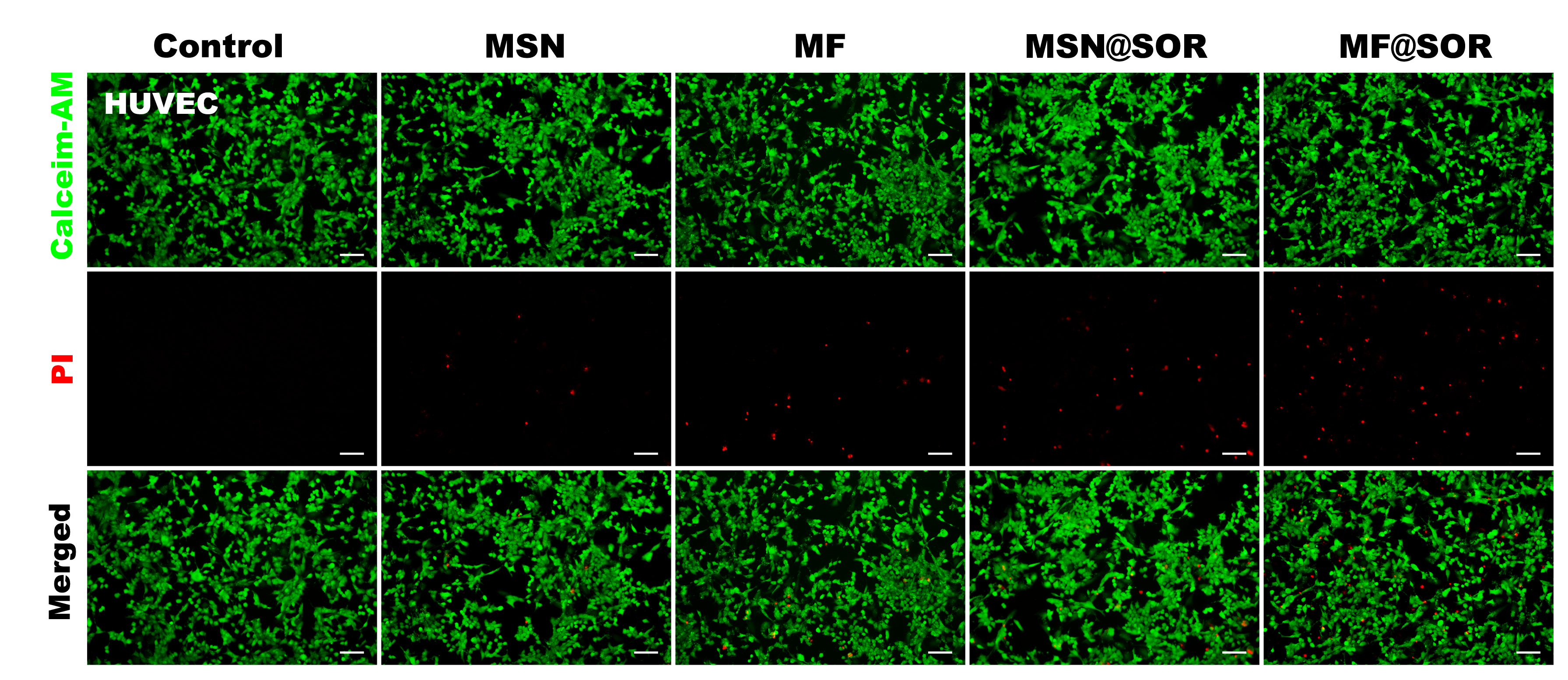


**Figure S7**. CLSM images of live and dead HUVECs after incubation with different NPs (scale bars: 100 μm).


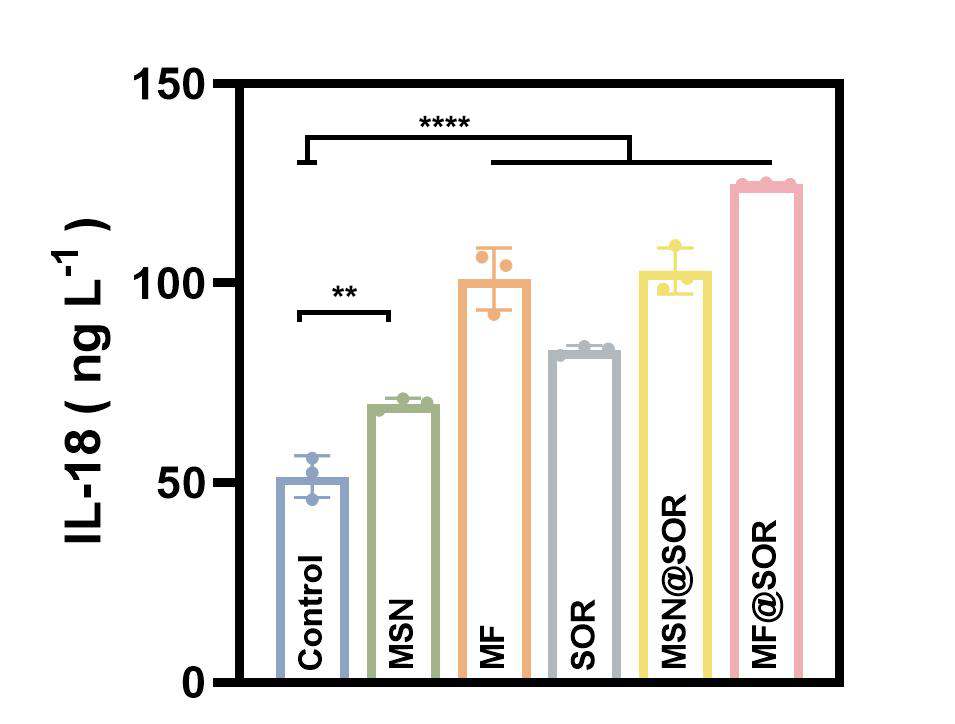


**Figure S8**. The levels of IL-18 measured in the supernatant after various treatments (n = 3). Data are represented as mean ± SD. All the p values were calculated by ANOVA. * *p* < 0.05, ** *p* < 0.01, *** *p* < 0.001, **** *p* < 0.0001, ns, not significant.


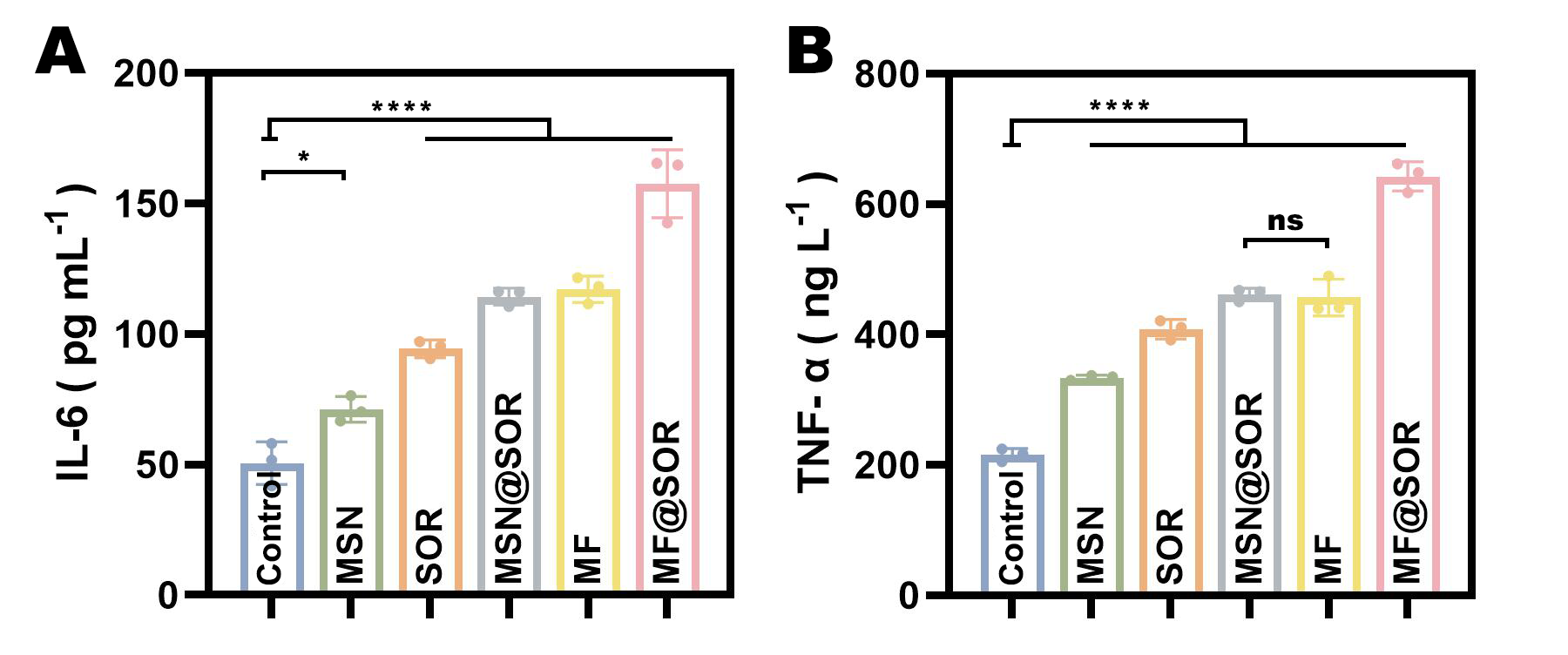


**Figure S9**. The concentrations of IL-6 (A) and TNF-α (B) quantified in the coculture system following different treatments (n = 3). Data are represented as mean ± SD. All the p values were calculated by ANOVA. * *p* < 0.05, ** *p* < 0.01, *** *p* < 0.001, **** *p* < 0.0001, ns, not significant.


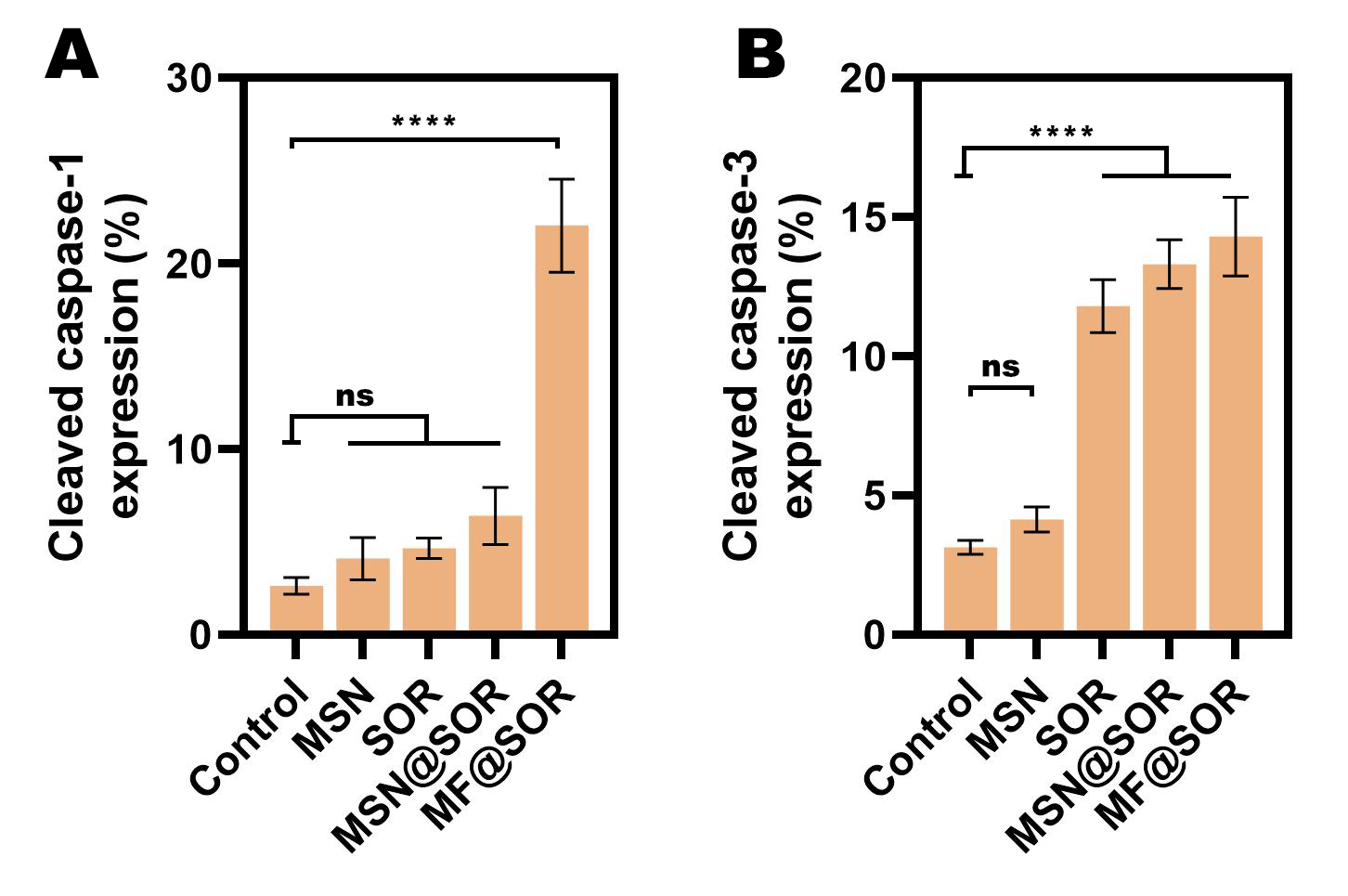


**Figure S10**. The quantitative analysis of (A) cleaved caspase-1 and (B) cleaved caspase-3 expression in hepa1-6 tumor upon different treatments. Data are represented as mean ± SD (n = 3). Data are represented as mean ± SD. All the p values were calculated by ANOVA. * *p* < 0.05, ** *p* < 0.01, *** *p* < 0.001, **** *p* < 0.0001, ns, not significant.


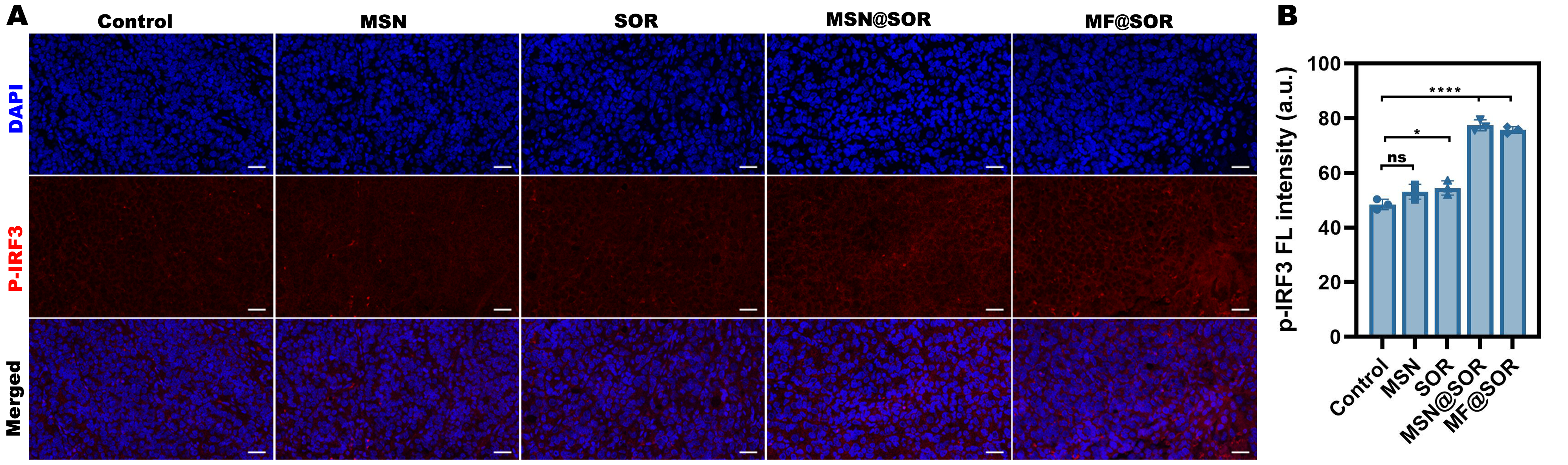


**Figure S11**. Immunofluorescence imaging of (A) p-IRF3 expression in hepa1-6 tumor upon different treatments (scale bar: 25 μ m) and (B) the corresponding semi-quantitative FL intensity analysis (n = 3). Data are represented as mean ± SD. All the p values were calculated by ANOVA. * *p* < 0.05, ** *p* < 0.01, *** *p* < 0.001, **** *p* < 0.0001, ns, not significant.


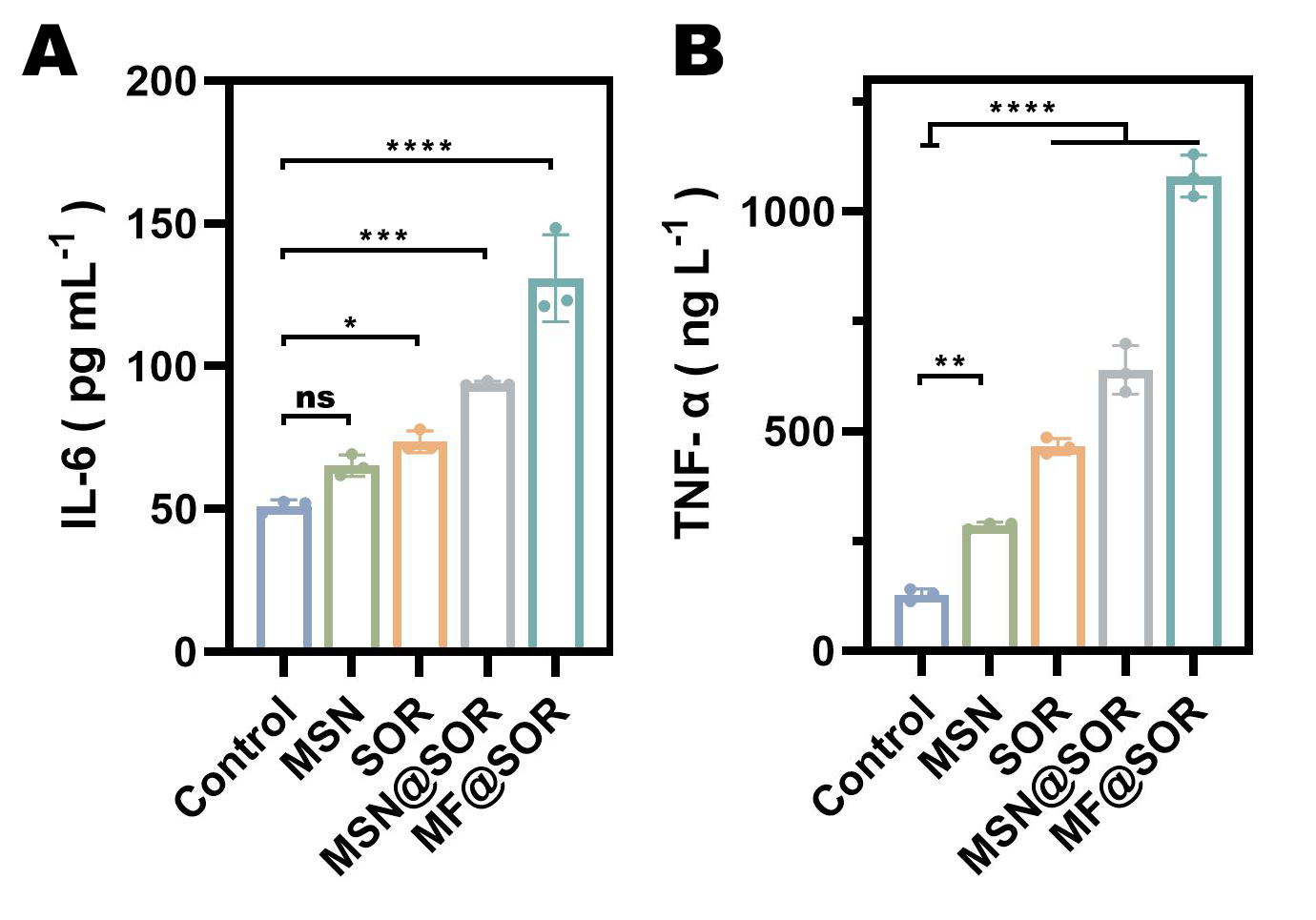


**Figure S12**. The level of (A) IL-6 and (B) TNF-α measured in mice serum after various treatments (n = 3). Data are represented as mean ± SD. All the p values were calculated by ANOVA. * *p* < 0.05, ** *p* < 0.01, *** *p* < 0.001, **** *p* < 0.0001, ns, not significant.


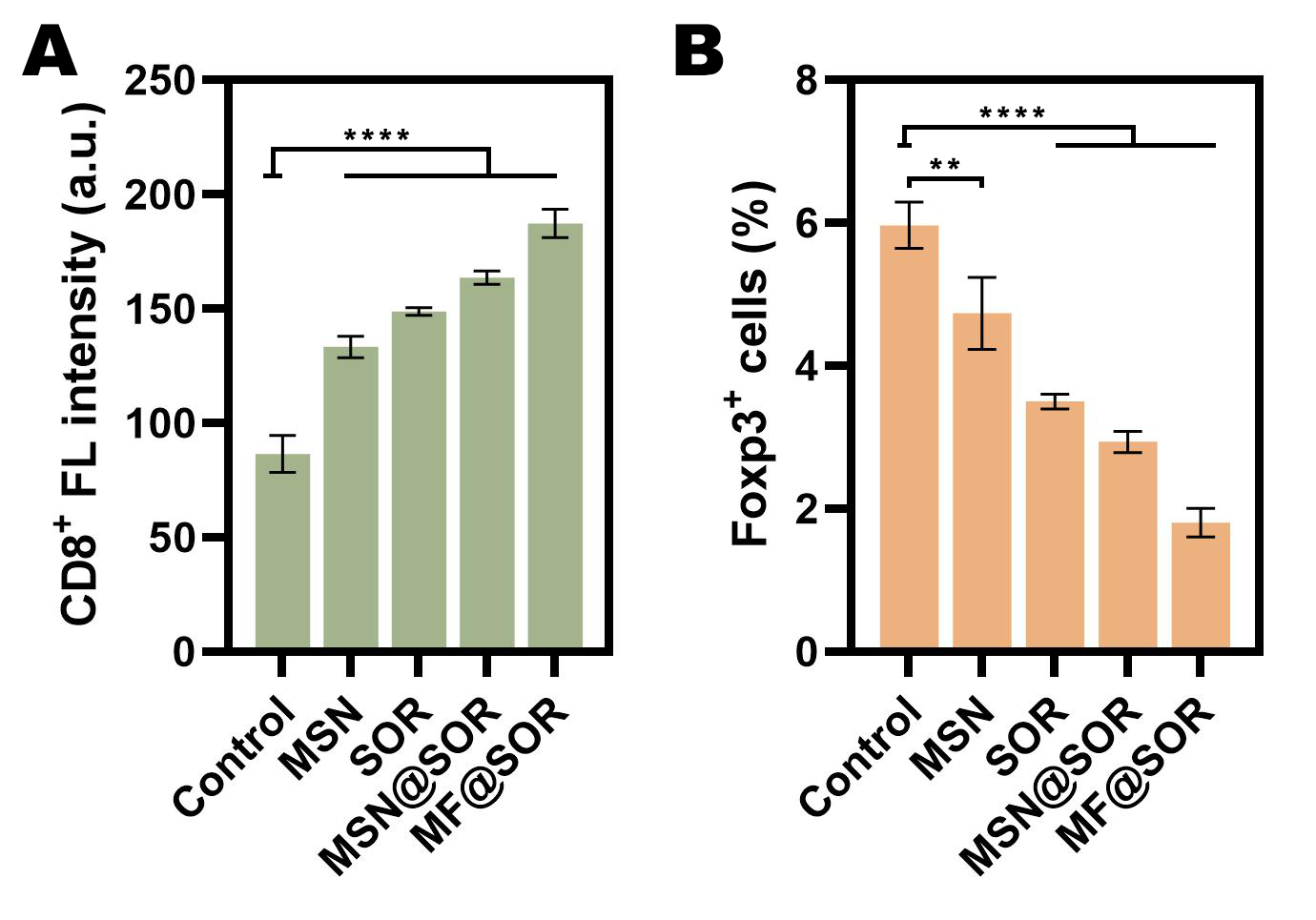


**Figure S13.** The quantitative analysis of (A) CD8^+^ T cells and (B) Foxp3^+^ expression in hepa1-6 tumor upon different treatments. Data are represented as mean ± SD (n = 3). All the p values were calculated by ANOVA. * *p* < 0.05, ** *p* < 0.01, *** *p* < 0.001, **** *p* < 0.0001, ns, not significant.


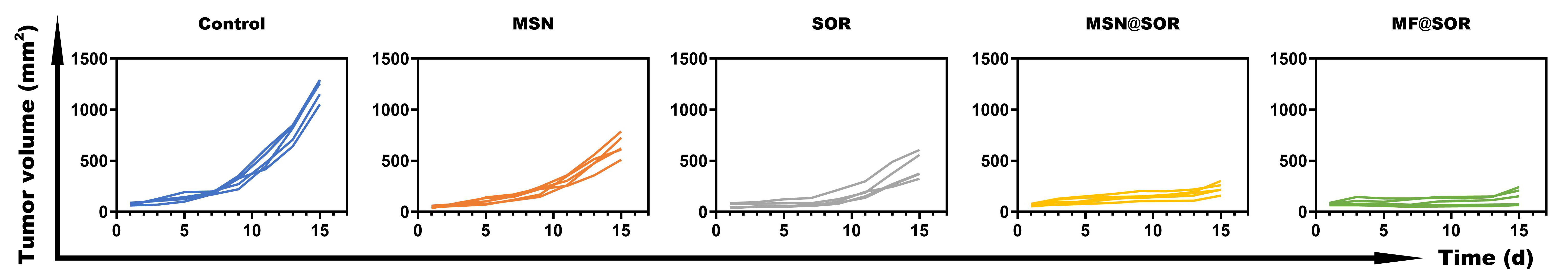


**Figure S14**. Tumor growth curves of individual mouse in each group.


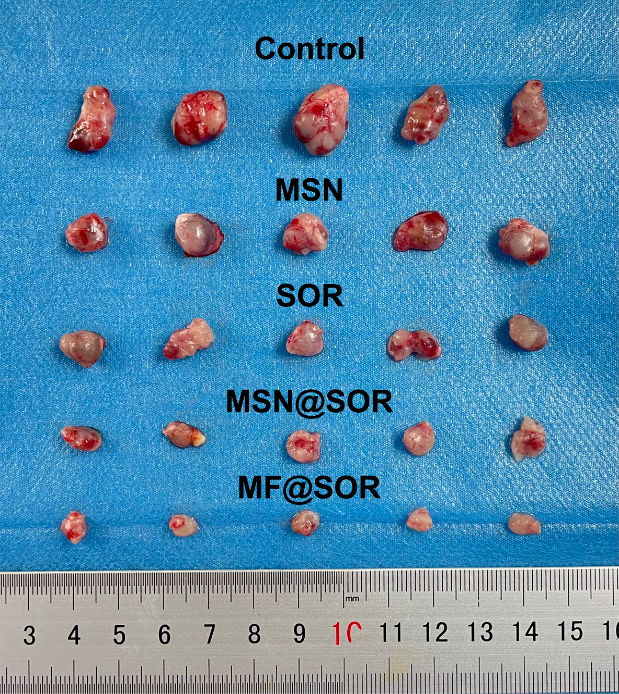


**Figure S15.** Digital photos of dissected tumors of tumor recurrence models in each group.


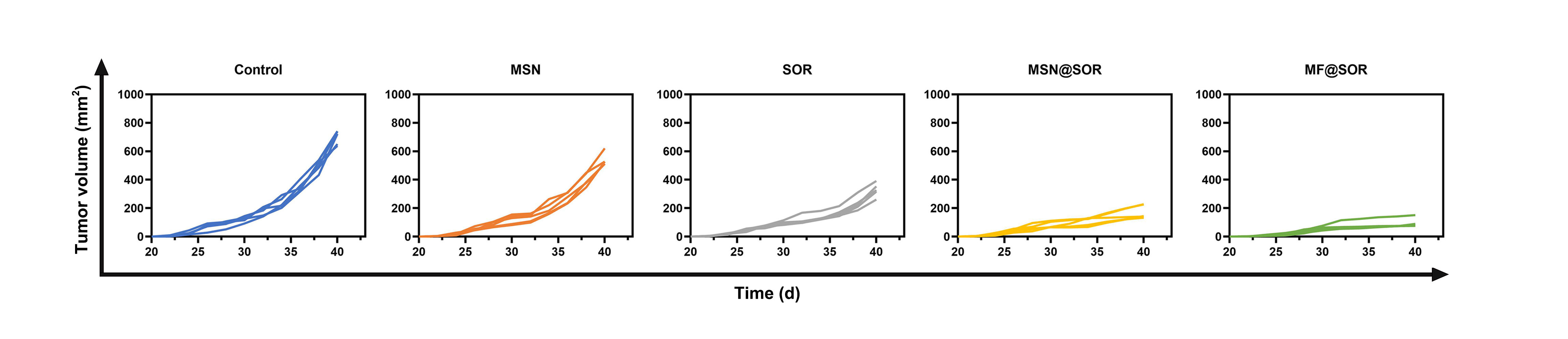
 **Figure S16.** Tumor growth curves of individual mouse in tumor recurrence models.


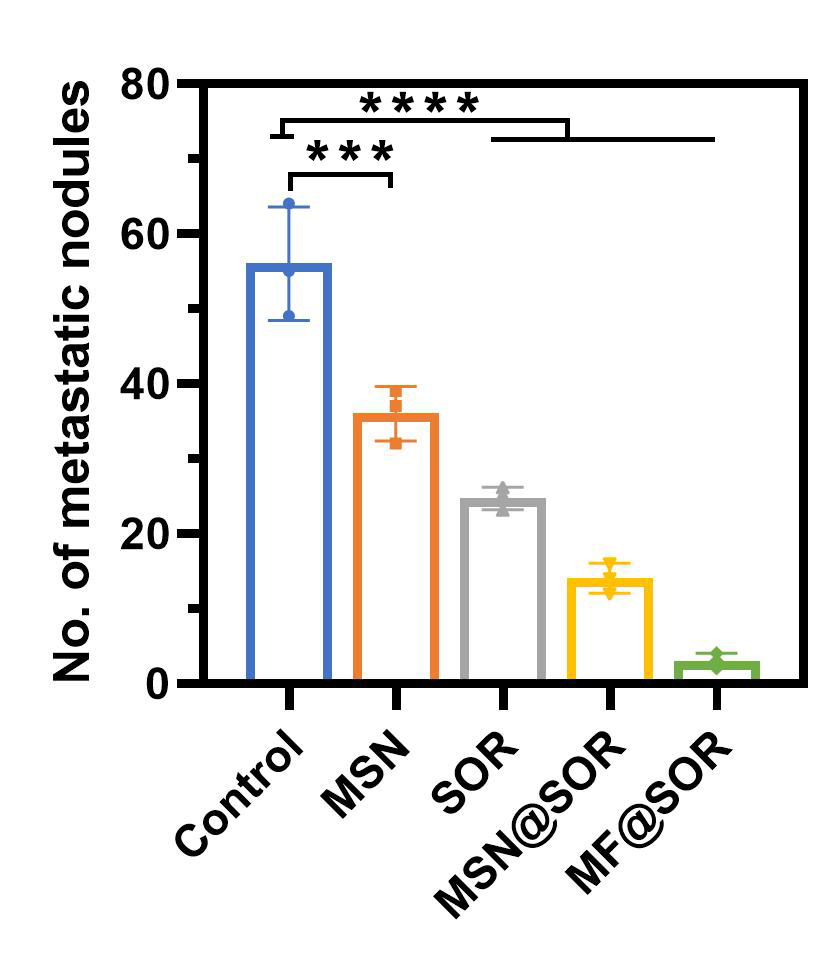


**Figure S17.** Quantitative analysis about the tumor nodules in lungs. Data are represented as mean ± SD (n = 3). All the p values were calculated by ANOVA. * *p* < 0.05, ** *p* < 0.01, *** *p* < 0.001, **** *p* < 0.0001, ns, not significant.


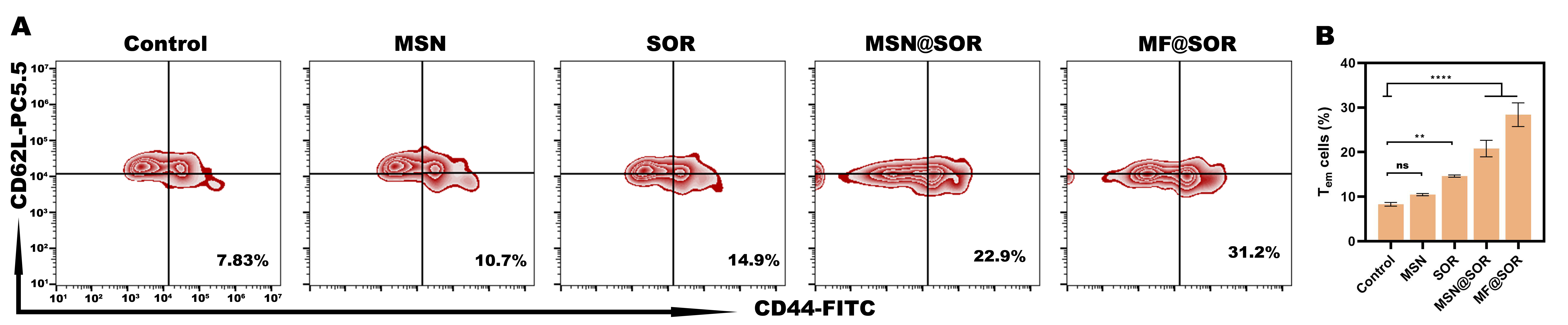


**Figure S18.** (A) The representative FCM images of the T_em_ cells (CD3^+^ CD8^+^ CD44^+^ CD62L^-^) proportion within the spleens in each group and (B) the quantative analysis (n = 3). Data are represented as mean ± SD. All the p values were calculated by ANOVA. * *p* < 0.05, ** *p* < 0.01, *** *p* < 0.001, **** *p* < 0.0001, ns, not significant.


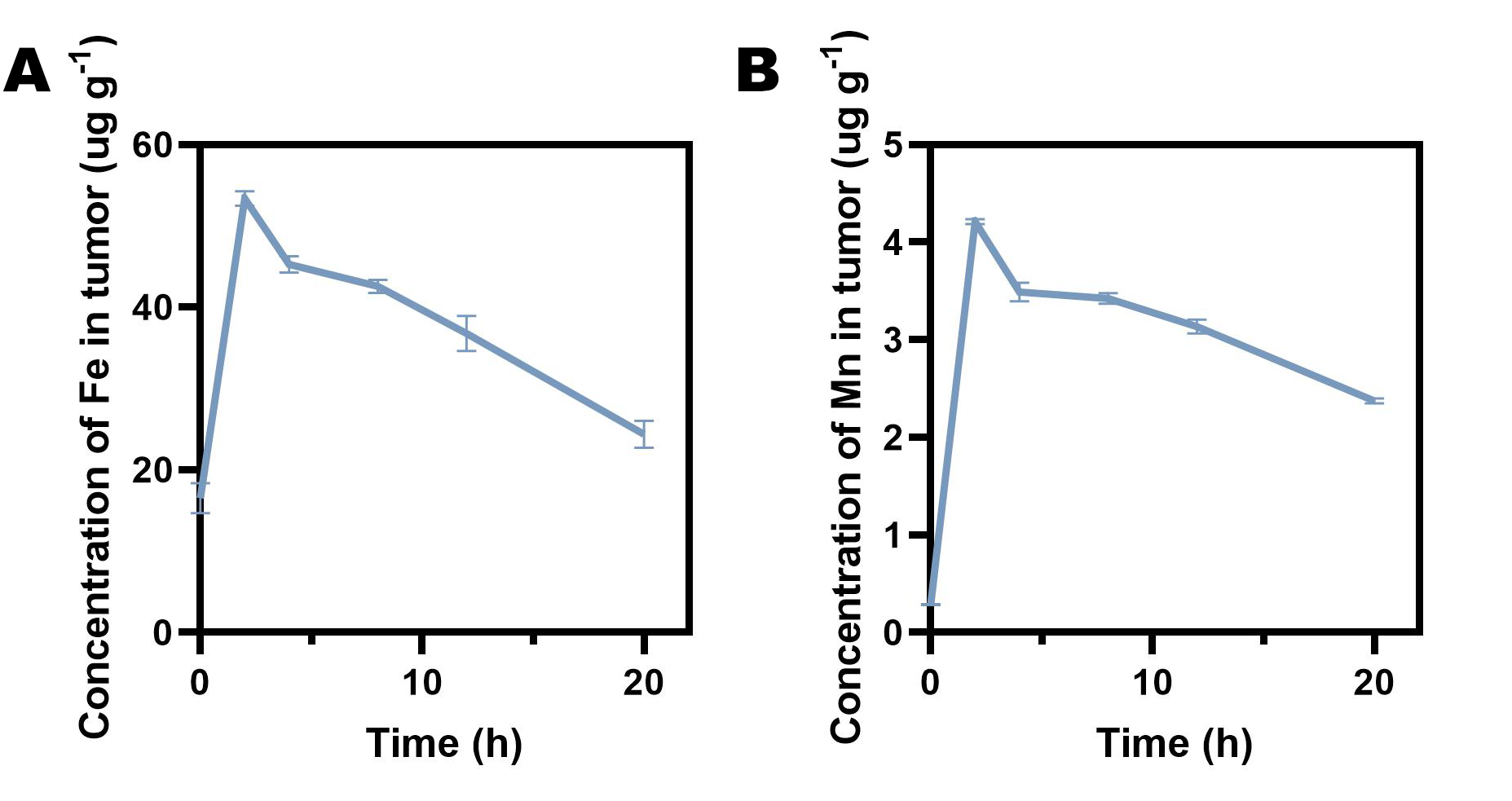


**Figure S19.** ICP-MS analysis of Fe (A) and Mn (B) in tumors at different time intervals post the MF@SOR injection. Data are represented as mean ± SD (n = 3).


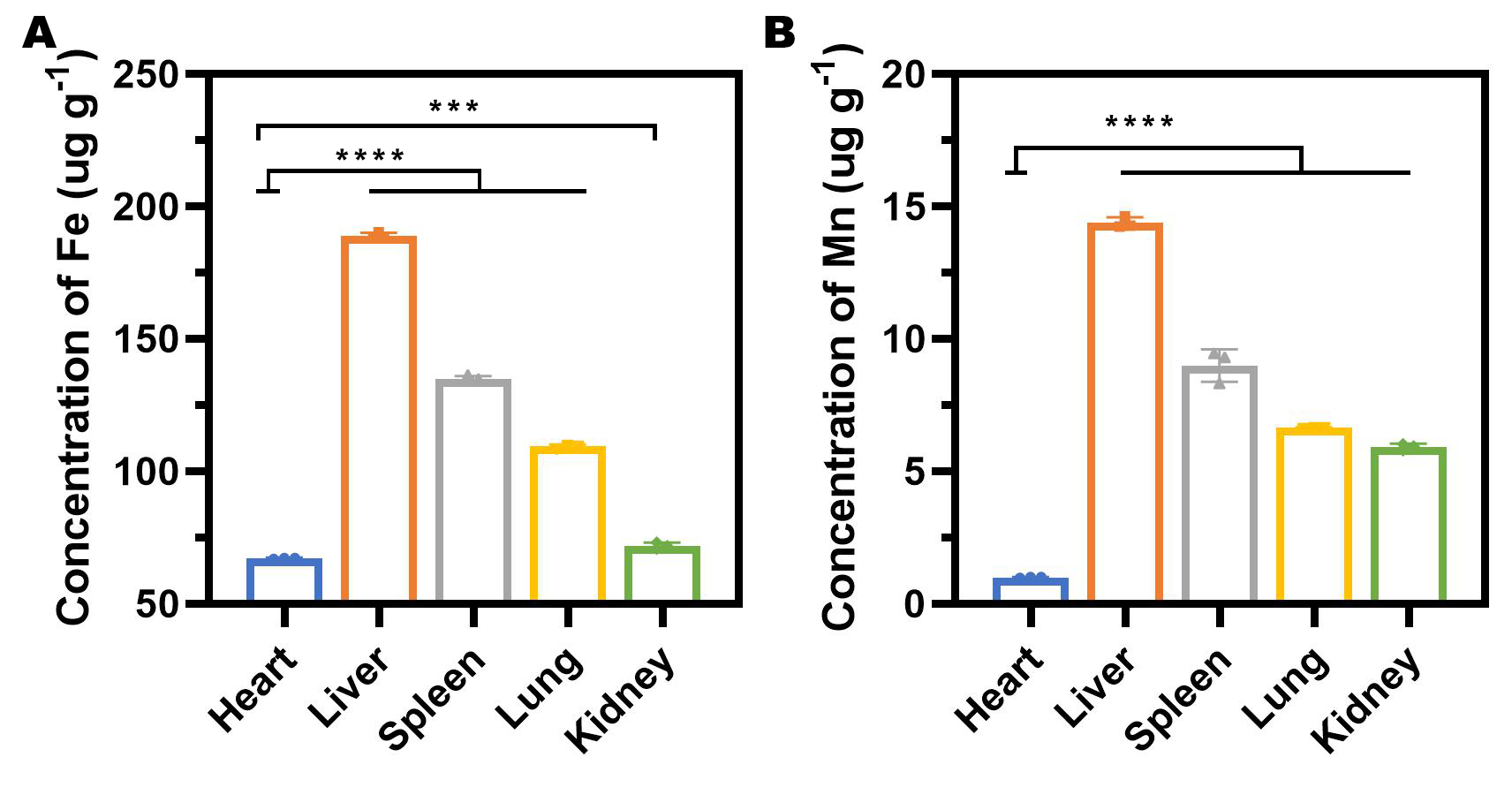


**Figure S20.** ICP-MS analysis of Fe (A) and Mn (B) in various organs post-treatment. Data are represented as mean ± SD (n = 3).


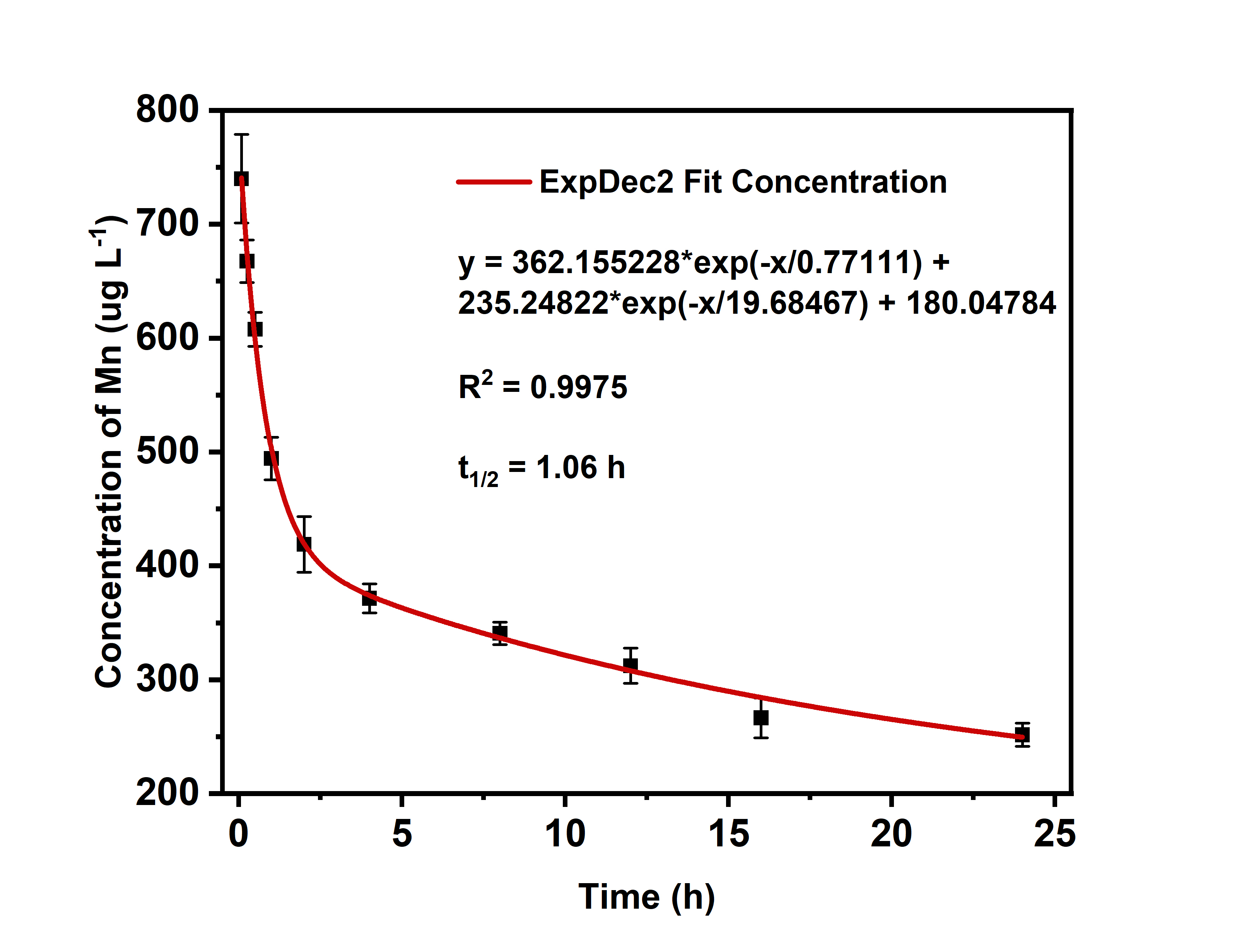


**Figure S21.** ICP-MS analysis of Mn in the blood at different time intervals post the MF@SOR injection. Data are represented as mean ± SD (n = 3).


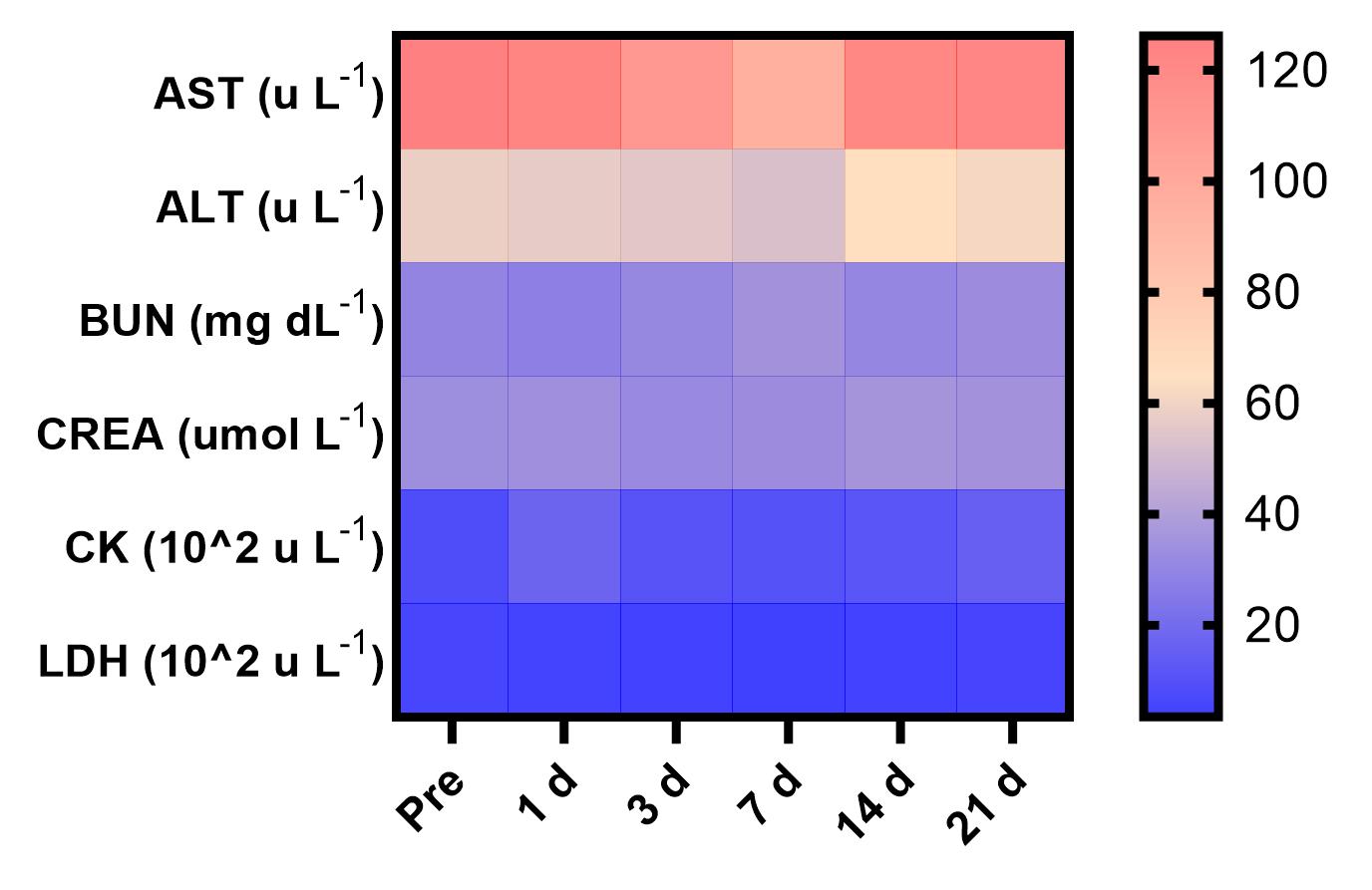


**Figure S22.** Blood biochemical examination of Kunming mice after intravenous injection of the MF@SOR NPs at different time points.
